# From preconception risk to perinatal outcomes: machine learning prediction of depression and acute psychiatric care from electronic health records in the UK

**DOI:** 10.1136/bmjment-2026-302945

**Published:** 2026-11-10

**Authors:** Benedetta Vai, Federico Calesella, Christoph Mueller, Chiara Verga, Edoardo Venturelli, Alessandra Biaggi, Claudio Singh Solorzano, Maria Grazia Di Benedetto, Kristi Priestley, Clare Taylor, Riddhi Laijawala, Annamaria Cattaneo, Robert Stewart, Francesco Benedetti, Carmine M Pariante

**Affiliations:** 1 Unit of Psychiatry and Clinical Psychobiology, IRCCS Ospedale San Raffaele, Milan, Italy; 2 Vita-Salute San Raffaele University, Milan, Italy; 3 Department of Psychological Medicine, Institute of Psychiatry, Psychology & Neuroscience, King’s College London, London, UK; 4 South London and Maudsley NHS Foundation Trust, London, UK; 5 Department of Pharmacological and Biomolecular Sciences, University of Milan, Milan, Italy; 6 Biological Psychiatry Unit, IRCCS Istituto Centro San Giovanni di Dio Fatebenefratelli, Brescia, Italy; 7 Department of Psychological Medicine, King’s College London Institute of Psychiatry Psychology & Neuroscience, London, UK; 8 National Institute for Health and Care Research (NIHR), Maudsley Biomedical Research Centre at South London and Maudsley NHS Foundation Trust and King’s College London, London, UK

**Keywords:** Affective Disorders, Psychotic, Depressive Disorder, Mental Health Services

## Abstract

**Background:**

Perinatal depression is common among women with pre-existing psychiatric disorders and may require acute psychiatric care yet tools for early risk stratification remain limited.

**Objective:**

To investigate whether routinely collected pre-conception electronic health record data can predict perinatal depressive episodes and the need for acute psychiatric admission or home treatment among women receiving secondary mental healthcare.

**Methods:**

This retrospective cohort included 20 793 pregnancies among 14 134 women with an active referral to secondary mental health services and deliveries between 2012 and 2020. Using the Biomedical Research Centre Case Register, we extracted preconception sociodemographic, lifestyle, reproductive, clinical, treatment-related, psychiatric and medical comorbidity variables. Pregnancy-related and birth-related predictors were additionally included for postnatal outcomes. Elastic net-penalised logistic regression models were developed to predict depressive episodes and acute psychiatric admission or home treatment during the overall perinatal, antenatal and postnatal periods.

**Findings:**

Approximately 16% of pregnancies were associated with a perinatal depressive episode, of which approximately 19% involved acute psychiatric admission or home treatment. Models predicting depressive episodes achieved moderate discrimination across perinatal periods (area under the receiver operating characteristic curve (AUC) 0.67–0.68; sensitivity 61%–63%; specificity 72%–75%). Key predictors included greater preconception depressive and positive symptom severity, previous antidepressant, antipsychotic and mood stabiliser use, primiparity and greater socioeconomic deprivation. Smoking during pregnancy was additionally associated with postnatal depression. Models predicting acute psychiatric admission or home treatment were significant for the overall perinatal and postnatal periods but showed lower discrimination (AUC 0.63; sensitivity 42%; specificity 84%–85%). Key predictors included previous antipsychotic and hypnotic use and Black ethnicity.

**Conclusions:**

Routinely collected preconception electronic health record data can provide moderate discrimination of subsequent perinatal depression risk, whereas prediction of acute psychiatric deterioration remains limited.

**Clinical implications:**

Preconception clinical information may support early identification of women at increased risk of perinatal depression and inform targeted monitoring and preventive care. Prediction of acute psychiatric care needs is likely to require additional longitudinal and pregnancy-specific clinical information.

**WHAT IS ALREADY KNOWN ON THIS TOPIC**

- Perinatal depression is common among women with pre-existing psychiatric disorders and is associated with substantial maternal and child morbidity. Previous machine learning studies have shown moderate accuracy in predicting postpartum depression, but most focused on limited clinical variables, specific perinatal periods or general population samples. Furthermore, few studies have investigated the prediction of severe perinatal psychiatric outcomes requiring acute psychiatric admission or intensive home treatment.

**WHAT THIS STUDY ADDS**

- This study shows that routinely collected preconception electronic health record data can moderately predict the risk of perinatal depression across pregnancies among women with previous contact with secondary mental health services. Pre-existing affective and psychotic-spectrum symptom severity, previous psychotropic treatment, primiparity and socioeconomic disadvantage emerged as the most stable predictors across antenatal and postnatal periods. The study also provides one of the first machine learning investigations of acute psychiatric admission or home treatment during the perinatal period.

**HOW THIS STUDY MIGHT AFFECT RESEARCH, PRACTICE OR POLICY**

- These findings support the potential use of preconception electronic health records for early risk stratification and monitoring of pregnancies in women already engaged with mental health services. Integrating longitudinal symptom trajectories, psychosocial variables and biological markers may improve prediction of severe perinatal outcomes requiring acute psychiatric care. The results may inform the development of preventive and stratified perinatal mental health pathways within secondary care services.

## Introduction

 Pregnancy and the postnatal period represent phases of increased vulnerability for the onset and recurrence of psychiatric disorders, with the risk of depression up to twofold higher than in other periods of a woman’s life.^1^ The global prevalence of perinatal depression (PD) is approximately 17% in the general population, with higher rates reported in low and middle-income countries.^2^ The risk is further increased among women with pre-existing psychiatric conditions, including a history of major depressive disorder, bipolar disorder, obsessive–compulsive disorder or psychotic disorders.^2 3^ PD is also associated with significant morbidity for both mother and child, including increased risk of maternal suicide, a leading cause of maternal death in the first year after birth, and infanticide, disrupted mother–infant bonding and adverse cognitive and emotional development in offspring.^4^

Despite its clinical relevance and public health impact, a large proportion of women with PD do not receive clinical evaluation or treatment. Population-based data indicate that only approximately 30%–50% of women with clinically significant depressive symptoms are identified within healthcare settings, and fewer than 20% receive any form of intervention.^5^ Among those who access care, treatment allocation generally reflects symptom severity. Mild to moderate presentations are most often managed in outpatient settings with evidence-based psychotherapies combined with pharmacological treatments. A smaller proportion of women (0.1%–0.2%) develop severe postpartum episodes with psychotic features, a condition more closely aligned with bipolar disorder, characterised by marked functional impairment or elevated suicidal or infanticidal risk, requiring psychiatric admission, specialist mother-and-baby unit care or intensive home-treatment services.^6^ However, tools to identify women at risk of depressive onset and subsequent escalation to acute care remain limited.

Multiple sociodemographic, behavioural, obstetric and clinical factors are associated with increased risk of PD. Higher odds are observed among women with socioeconomic disadvantage, smoking, previous pregnancy loss or pregnancy-related complications such as gestational diabetes and obesity and previous psychiatric disorders.^7^ Additional vulnerability factors include trauma exposure, intimate partner violence, limited social support and poor marital satisfaction.^8^ Collectively, these prepregnancy characteristics provide a clinically relevant framework for risk stratification and preventive interventions within routine maternity care.

In this perspective, machine learning (ML) approaches provide a data-driven framework to integrate complex sociodemographic, clinical, obstetric and psychosocial information and generate individualised, pregnancy-specific risk predictions that may not be captured by traditional statistical methods.^9^ Previous ML studies have shown moderate predictive accuracy for PD, particularly for postnatal depression onset (area under the receiver operating characteristic curve (AUC)≈0.70), identifying predictors such as prior depression or anxiety, psychosocial stress, low social support and selected obstetric or medical factors.^10^ However, most studies focused on specific trimesters or restricted perinatal phases and lacked a detailed characterisation of preconception psychiatric and medical morbidity. Furthermore, to the best of our knowledge, no studies have addressed whether acute psychiatric admission or home-treatment use can be predicted within depressed women, which could support the early identification of women at higher risk of severe psychiatric outcomes.

In the present study, we applied an elastic net ML approach within a large secondary mental healthcare cohort in the United Kingdom to predict, at the pregnancy level, (1) the onset of PD and (2) admission to acute psychiatric care or home-treatment services. We used electronic health records (EHR) data including sociodemographic, lifestyle, reproductive, clinical, treatment-related and International Classification of Diseases (ICD)-10-derived psychiatric and medical comorbidity variables. For postnatal prediction, pregnancy-related and birth-related factors, including smoking during pregnancy, season of delivery, obstetric interventions and preterm birth, were additionally considered. Analyses were performed for the overall perinatal period and separately for antenatal and postnatal outcomes. By focusing on women with an active referral to secondary mental health services on or before delivery, the study examined prediction in a clinically vulnerable population with well-characterised psychiatric histories and elevated risk of depression and severe mental health outcomes.

## Methods

### Cohort description

We conducted an observational retrospective cohort study implementing an elastic net ML approach to predict the (1) onset of depression and the (2) admission to acute psychiatric care or home treatment. The sample comprised pregnancies identified through NHS hospital episodes documenting a birth between 2012 and 2020. Eligible pregnancies were those in women who had an active referral to South London and Maudsley (SLaM) NHS Foundation Trust mental health services on or before the delivery date (EPISTART).^11^ Pregnancy was the unit of analysis, and women could contribute more than one eligible pregnancy, each characterised by its corresponding preconception history and pregnancy-specific factors and outcomes. SLaM provides secondary and specialist mental healthcare to a catchment population of approximately 1.2 million residents across four South London boroughs. All clinical care within SLaM has been documented using fully EHR since 2006. These records underpin the South London and Maudsley Biomedical Research Centre Case Register, a large-scale longitudinal database containing comprehensive clinical information on all individuals receiving care from SLaM services. Access to the Case Register is provided via the Clinical Record Interactive Search (CRIS) platform,^12^ which enables secure extraction of deidentified data derived from both structured fields and unstructured free-text clinical notes. CRIS has been enhanced through a range of natural language processing (NLP) algorithms applied to text fields to extract metadata on entities such as diagnoses, medication and a range of psychiatric symptoms,^12 13^ Maudsley Biomedical Research Centre, Natural Language Processing Service 2020. Available from: https://www.maudsleybrc.nihr.ac.uk/facilities/clinical-record-interactive-search-cris/natural-language-processing-nlp-service/. In addition, CRIS has been successfully linked to a range of external data resources including Hospital Episode Statistics (HES), which provide statistical data on all NHS hospital care in England, including childbirths.^11^

### Ethical consideration

Ethical approval was granted for CRIS as a source of data for secondary analysis by the Oxfordshire Research Ethics Committee C (reference: 23/SC/0257) without participants’ informed consent being required for its anonymous nature of data.

### Patients and public involvement

Patients and members of the public were not involved in the design, conduct, reporting or dissemination plans of this research.

### Outcome variables

Cases were defined as pregnancy instances associated with a depressive episode in the antenatal and/or postnatal period. The linkage with HES was used to identify pregnancy instances: delivery episodes were identified from hospital records reporting deliveries occurring after 24 weeks of gestation.^11^ Delivery date was used as the temporal anchor, with conception estimated by subtracting 270 days. Three periods were then defined: period 1—preconception: spanning the 2 years prior to conception, excluding the conception date; period 2—antenatal, spanning conception to birth, including both conception and birth and period 3—postnatal, spanning birth to 1 year after birth, excluding the birth date. The postpartum period was extended to 1 year after delivery to capture clinically relevant depressive episodes that may emerge several months after childbirth.^14^ Pregnancy instances from women who died during the postnatal follow-up period were excluded because the full postnatal observation window could not be completed; this only occurred in 7 of the 14 134 unique women represented in the cohort. Depressive episodes within these periods were identified either through the presence of four or more of 21 possible depressive symptoms ascertained via the CRIS NLP Applications Library (see online supplemental method 1), or through ICD diagnostic codes for depression, including bipolar depression and PD in CRIS (F31.3, F31.4, F31.5, F32–F33, F53). The CRIS NLP applications were previously validated against expert manual annotation, achieving a median F1 score of 0.88 across 46 psychiatric symptom extraction models^13^ (see online supplemental method 2). Pregnancy instances without depressive symptoms or diagnostic codes for depression during either period 2 or period 3 were classified as controls. For analyses predicting admission to acute psychiatric care or home treatment, both cases and controls were restricted to pregnancy instances associated with PD during period 2 and/or period 3. Acute care was defined as either an inpatient ward or home treatment in CRIS, which includes care at home by staff available every day, who can visit up to three times per day.^11^ In these models, cases were defined as pregnancy instances associated with the specific outcome during the relevant time-window (period 2 and/or period 3), while controls were pregnancy instances not associated with the outcome during the corresponding period.

### Predictor variables

In all analyses, preconception variables (period 1) and sociodemographic variables were entered as predictors (see online supplemental table 1). Sociodemographic variables (age; ethnicity; marital status; Index of Multiple Deprivation, IMD,^15^ an area-level measure of socioeconomic deprivation. Health-related lifestyle factors (smoking), reproductive and pregnancy-related characteristics (number of previous pregnancies, primiparity), clinical symptom severity measures were extracted with CRIS NLP (depressive, manic, positive, negative, disorganised, catatonic and other symptom scores)^13^ and previous treatments (eg, cognitive behavioural therapy, family interventions, antipsychotics, antidepressants, mood stabilisers, hypnotics) were indexed for period 1. Previous medical and psychiatric comorbidities (period 1) were identified using ICD-10 diagnostic codes derived from linked HES and CRIS records. ICD-10 macrocategories were entered as predictors, alongside more specific second-level groupings for mental and neurodevelopmental disorders, pregnancy-related, childbirth-related and puerperium-related conditions, and factors influencing health status and contact with health services. In addition, selected third-level subcodes capturing clinically relevant conditions associated with PD were included, namely obesity, puerperium-related mental disorders and borderline personality disorder (see online supplemental method 2). All ICD-10 variables were coded as binary indicators reflecting the presence or absence of diagnoses prior to conception. Sixty-nine predictors were added for models in peripartum and antenatal period. For postnatal outcome models, additional predictors extracted during pregnancy (period 2) were included, namely smoking, season of delivery, obstetric interventions (ie, episiotomy, caesarean section, forceps delivery and anaesthetic procedures) and preterm birth, resulted in 76 predictors for postnatal period. Preterm birth was defined as delivery before 37 weeks of gestation based on recorded gestational age or ICD-10 delivery codes from HES records.^11^

### Statistical analyses

Descriptive statistics were performed comparing pregnancy instances with and without PD. χ^2^ tests and t-tests were used to compare these groups. Results were corrected for multiple comparisons using false discovery rate (FDR). Missing data were initially imputed using a K-nearest neighbours approach with hyperparameter optimisation (see online supplemental method 3). Data were subsequently incorporated into a predictive pipeline consisting of feature standardisation followed by elastic net–penalised logistic regressions. Models were designed to predict the (1) onset of depression and the (2) admission to acute psychiatric care or home treatment. Analyses were performed covering the whole perinatal period (periods 2 and 3) and stratified for antenatal (ie, period 2) or postnatal (period 3) ones, resulting in a total of six models. Categorical variables with more than two levels, including ethnicity, marital status and season of delivery, were entered into the models using one-hot encoding. Models’ evaluation was conducted using a fivefold nested cross-validation (CV) scheme (see online supplemental method 4), where standardisation, hyperparameter optimisation and model fitting were performed within each scheme. CV folds were stratified to preserve the class distribution, and the model’s loss function was reweighed to penalise the majority class proportionally to its imbalance relative to the minority class. Cross-validated predictions were used to compute performance metrics, including accuracy, balanced accuracy (BA), F1 score, sensitivity, specificity, positive predictive value (PPV), negative predictive value and AUC. Statistical significance was assessed using permutation testing with 5000 iterations and a Bonferroni-corrected threshold of p<0.008. To identify predictors contributing significantly to the models, bootstrap resampling with bias-corrected and accelerated (BCa) CIs was performed. Variable inclusion probabilities were additionally calculated to estimate predictor stability across resampling iterations. Given the class imbalance between pregnancy instances with and without depression, supplementary analyses were performed in which the majority class (eg, pregnancies without depression) was randomly downsampled to match the size of the minority class (eg, pregnancies with depression). The full pipeline was executed on both the whole and downsampled datasets and stratified for perinatal periods.

## Results

The sample included 20 793 pregnancies from 14 134 women (table 1), of which 3305 (15.9%) were associated with PD; 1915 (9.2%) included an antenatal depressive episode and 2197 (10.6%) a postnatal depressive episode. Among the 14 134 women included in the cohort, 8914 (63.07%) contributed with one eligible pregnancy, 4079 (28.86%) with two, 920 (6.51%) with three, 167 (1.18%) with four and 54 (0.38%) with five or more pregnancies. Among pregnancies with PD, 621 (18.8%) involved acute psychiatric admission or home treatment during the perinatal period, including 223 (12.3%) antenatally and 465 (21.7%) postnatally. Pregnancies associated with PD were more frequent among women from black and other minority ethnic groups, those experiencing greater socioeconomic disadvantage and those with smoking during pregnancy, primiparity, greater preconception psychiatric symptom severity, previous psychotropic treatment and psychiatric comorbidity (online supplemental table 2).

**Table 1:** Characteristics of pregnancy instances with and without perinatal depression

	Perinatal depression, n=3305	No perinatal depression, n=17 488	t/χ2	Cohen’s d/Cramer’s V	P value
Preconception indexed variables					
Age	30.83±6.20	30.85±6.01	0.17	0.003	0.87
Ethnicity White British and other White	1669 (50.5%)	10 661 (61.0%)	133.03	0.08	<0.001*
Black African and other Black	1037 (31.4%)	4545 (26.0%)			
Asian/Mixed/Other	599 (18.1%)	2282 (13.0%)			
Marital status					
Single	1617 (48.9%)	11 721 (67.0%)	544.66	0.16	<0.001*
cohabiting	582 (17.6%)	2793 (16.0%)			
Married	969 (29.3%)	2488 (14.2%)			
Separated/divorced/widowed	137 (4.2%)	486 (2.8%)			
Index of multiple deprivation	26.49±9.44	25.78±10.47	−3.59	−0.07	<0.001*
Number of previous pregnancies	1.10±1.39	1.35±1.48	9.1	0.17	<0.001*
Primiparity	1452 (43.9%)/1853 (56.1%)	5954 (34.0%)/11 534 (66.0%)	118.07	0.08	<0.001*
Cognitive behavioural therapy/family interventions	119 (3.6%)/3186 (96.4%)	275 (1.6%)/17 213 (98.4%)	60.42	0.05	<0.001*
Use of antipsychotics	460 (13.9%)/2845 (86.1%)	520 (3.0%)/16 968 (97.0%)	738.99	0.19	<0.001*
Use of antidepressants	769 (23.3%)/2536 (76.7%)	1434 (8.2%)/16 054 (91.8%)	664.66	0.18	<0.001*
Use of mood stabilisers	183 (5.5%)/3122 (94.5%)	188 (1.1%)/17 300 (98.9%)	313.27	0.12	<0.001*
Use of hypnotics	395 (12.0%)/2910 (88.0%)	557 (3.2%)/16 931 (96.8%)	486.97	0.15	<0.001*
Depressive symptom score	1.84±3.07	0.58±1.58	-34.89	0.66	<0.001*
Manic symptom score	0.99±1.74	0.31±0.87	-33.63	0.64	<0.001*
Positive symptom score	1.10±2.39	0.27±1.04	-32.54	0.62	<0.001*
Negative symptom score	0.44±1.01	0.09±0.44	-31.5	0.60	<0.001*
Disorganised symptom score	0.33±0.87	0.08±0.36	-27.39	0.52	<0.001*
Catatonic symptom score	0.03±0.19	0.006±0.08	-13	0.25	<0.001*
Other symptoms score	0.99±1.49	0.39±0.90	-30.85	0.59	<0.001*
Perinatal indexed variables					
Season of delivery					
Spring	858 (26.0%)	4499 (25.7%)	0.73	0.01	0.87
Summer	843 (25.5%)	4400 (25.2%)			
Autumn	823 (24.9%)	4338 (24.8%)			
Winter	781 (23.6%)	4251 (24.3%)			
Smoking	373 (11.3%)/2932 (88.7%)	925 (5.3%)/16 563 (94.7%)	169.76	0.09	<0.001*
Preterm birth	411 (12.4%)/2894 (87.6%)	2140 (12.2%)/15 348 (87.8%)	0.084	0.002	0.77
Birth with medical or obstetric intervention	2403 (72.7%)/902 (27.3%)	12 814 (73.3%)/4674 (26.7%)	0.42	0.01	0.51

Continuous variables are presented as mean±SD, or counts and percentages. Between-group comparisons were performed using t-tests or χ² tests, as appropriate, and corresponding p values and effect sizes are reported.

*P value significant after FDR correction.

FDR, false discovery rate.

ML models predicted PD onset with significant discrimination (all p<0.001, Bonferroni-corrected). AUC values approaching 0.68 indicated moderate discriminative ability, with sensitivity ranging from 61% to 63% and specificity from 72% to 75%, indicating a greater ability to correctly identify pregnancy instances without a depressive episode than those with a depressive episode. However, relatively low F1 scores (0.30–0.40) and PPVs (20–30%) indicated limited performance in correctly identifying pregnancies associated with a depressive episode (table 2). Bootstrap analyses identified multiple predictors within each of the time-window considered in the analysis, that is, perinatal, antenatal and postnatal. Specifically, a higher probability of developing PD (table 3, figure 1) was associated with greater depressive and positive symptom severity, primiparity, previous antidepressant, antipsychotic and mood stabiliser use, higher IMD, being married (vs single/cohabiting/separated/divorced/widowed), history of psychotic disorder (F20–F29), depressive episode (F32–F33), borderline personality disorder (F60.3) and other pregnancy-related disorders (O20-O29). Conversely, a lower probability of developing PD was associated with age, white ethnicity (vs other ethnic groups), single marital status (vs cohabiting/married/separated/divorced/widowed), number of previous pregnancies and selected preconception psychiatric diagnoses, including anxiety and stress-related disorders (F40–F48), behavioural syndromes associated with physiological disturbances (F50–F59), substance-use disorders (F10–F19), disorders with onset usually occurring in childhood and adolescence (F90–F98), and unspecified mental disorders (F99) as well as health hazard related to psycho-social and economic factors (Z55–Z65), and to family and personal history (Z77–Z99). The pattern of predictors for both antenatal and postnatal depression (online supplemental tables 3, 4, and figures 1, 2) largely overlapped with those observed for PD, with consistent associations with depressive symptom severity, previous antidepressant and mood stabiliser use, primiparity, marital status, ethnicity, IMD and psychiatric diagnostic categories, including substance-use, anxiety and stress-related disorders, behavioural syndromes (F10–F19, F40–F48, F50–F59). Positive associations with previous pregnancy-related maternal disorders (O20–O29) and psychotic disorder (F20–F29) were confirmed only in the antenatal period as well as negative associations with the number of previous pregnancies, and mental disorders in childhood and adolescence, or unspecified, and factors associated with personal or family history (F90–F98, F99, Z77–Z99) (online supplemental table 3). Associations similar in PD and in postnatal depression were found for antipsychotics and positive symptom score (online supplemental table 4). Antenatal depression showed additional positive and negative associations with metabolic disease (E00–E89), puerperium-related complications (O85–O92) and with other affective disorder (F34–F39) and complications during delivery (O60–O77) (online supplemental table 3). In contrast, postnatal depression was distinguished by a positive association with smoking during pregnancy and a negative association with factors related to health service contact (Z69–Z76) (online supplemental table 4).

**Figure 1:**
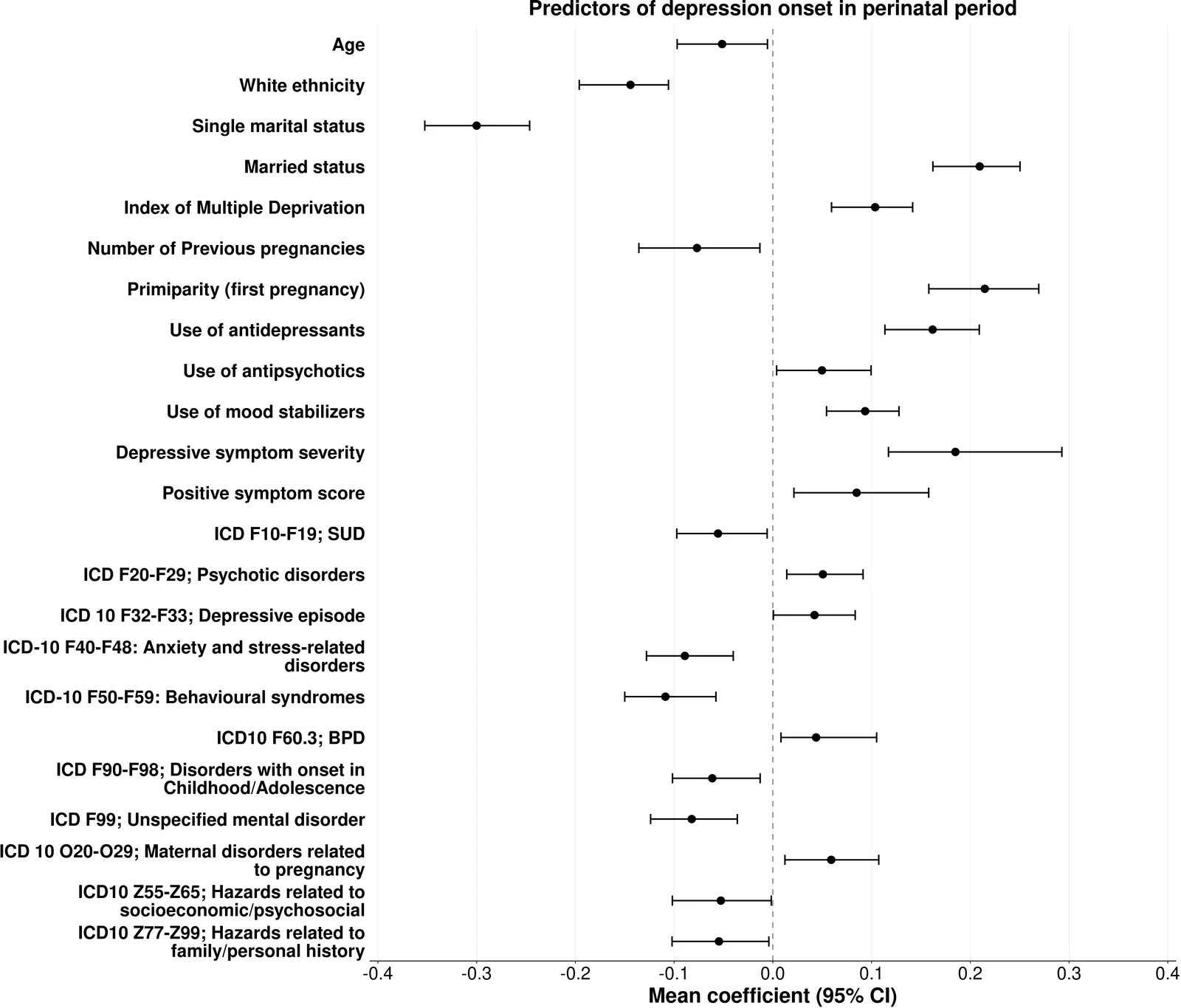
Forest plots of predictors of depression onset in perinatal period. Points indicate coefficient estimates in elastic net model and horizontal bars their 95% CIs. Positive coefficients denote association with increased likelihood of depression onset, whereas negative coefficients denote association with decreased likelihood. All variables were indexed before conception. BPD, Borderline Personality Disorder;ICD, International Classification of Diseases; SUD, Substance Use Disorders.

**Table 2:** Predictive performance of machine learning models for perinatal mental health outcomes across perinatal, antenatal and postnatal periods

	AUC	Accuracy	BA	F1	Sensitivity	Specificity	NPV	PPV	P value
Onset of depressive episodes									
Perinatal	0.67	70.35	67.17	40.13	62.51	71.83	91.02	29.55	<0.001
Antenatal	0.68	72.85	67.69	30.82	61.25	74.13	94.59	20.59	<0.001
Postnatal	0.68	73.32	68.43	34.2	62.13	74.73	94.01	23.60	<0.001
Admission to acute psychiatric care or home treatment									
Perinatal	0.63	75.70	62.58	39.12	41.55	83.61	86.08	36.96	<0.001
Antenatal	0.64	79.50	63.66	33.99	42.60	84.72	91.26	28.27	NS
Postnatal	0.63	75.50	63.37	42.62	41.94	84.80	84.05	43.33	<0.001

Performance metrics include area under the receiver operating characteristic curve (AUC), accuracy, balanced accuracy (BA), F1 score, negative predictive value (NPV), positive predictive value (PPV), sensitivity and specificity.

**Table 3:** Bootstrap estimates of preconceptional predictors associated with the onset of perinatal depression

Predictors	Bootstrap performance elastic net model			Bootstrap performance single logistic regression		
	Median β (95% CI)	OR (95% CI)	VIP	β (95% CI)	OR (95% CI)	P value
Age	−0.05 (−0.10 to −0.01)	0.95 (0.91 to 1.00)	98.9	−0.00 (−0.01 to 0.01)	1.00 (0.99 to 1.01)	0.87
White ethnicity (vs other ethnic groups)†	−0.15 (−0.20 to −0.11)	0.87 (0.82 to 0.90)	100	0.28 (0.23 to 0.33)	1.32 (1.26 to 1.39)	<0.001*
Single marital status (vs cohabiting/married)†	−0.30 (−0.35 to −0.25)	0.74 (0.70 to 0.78)	100	0.42 (0.39 to 0.46)	1.53 (1.47 to 1.59)	<0.001*
Married status (vs single/cohabiting)†	0.21 (0.16 to 0.25)	1.23 (1.18 to 1.28)	100			
Index of multiple deprivation	0.10 (0.06 to 0.14)	1.11 (1.06 to 1.15)	100	0.01 (0.00 to 0.01)	1.01 (1.00 to 1.01)	<0.001*
Number of previous pregnancies	−0.08 (−0.14 to −0.01)	0.93 (0.87 to 0.99)	99.48	−0.13 (−0.16 to −0.10)	0.88 (0.85 to 0.90)	<0.001*
Primiparity (first pregnancy)	0.21 (0.16 to 0.27)	1.24 (1.17 to 1.31)	100	0.42 (0.34 to 0.49)	1.52 (1.41 to 1.64)	<0.001*
Use of antidepressants	0.16 (0.11 to 0.21)	1.18 (1.12 to 1.23)	100	1.22 (1.12 to 1.32)	3.39 (3.08 to 3.74)	<0.001*
Use of antipsychotics	0.05 (0.00 to 0.10)	1.05 (1.00 to 1.10)	98.08	1.66 (1.53 to 1.80)	5.28 (4.62 to 6.02)	<0.001*
Use of mood stabilisers	0.09 (0.05 to 0.13)	1.10 (1.06 to 1.14)	100	1.69 (1.48 to 1.89)	5.39 (4.38 to 6.63)	<0.001*
Depressive symptom severity	0.19 (0.12 to 0.29)	1.20 (1.12 to 1.34)	100	0.24 (0.22 to 0.26)	1.27 (1.25 to 1.29)	<0.001*
Positive symptom score	0.09 (0.02 to 0.16)	1.09 (1.02 to 1.17)	99.16	0.30 (0.28 to 0.32)	1.35 (1.32 to 1.38)	<0.001*
ICD F10–F19; mental and behavioural disorders due to psychoactive substance use	−0.06 (−0.10 to −0.01)	0.95 (0.91 to 0.99)	99	0.07 (−0.18 to 0.32)	1.07 (0.83 to 1.38)	0.58
ICD F20–F29; schizophrenia, schizotypal, delusional, and other non-mood psychotic disorders	0.05 (0.01 to 0.09)	1.05 (1.01 to 1.10)	99.46	1.99 (1.73 to 2.25)	7.31 (5.62 to 9.51)	<0.001*
ICD 10 F32–F33; depressive episode	0.04 (0.01 to 0.08)	1.04 (1.00 to 1.09)	97.82	1.01 (0.88 to 1.15)	2.76 (2.41 to 3.15)	<0.001*
ICD-10 F40–F48: anxiety and stress-related disorders	−0.09 (−0.13 to −0.04)	0.92 (0.88 to 0.96)	100	0.25 (0.08 to 0.42)	1.29 (1.09 to 1.52)	<0.05*
ICD-10 F50–F59: behavioural syndromes associated with physiological disturbances	−0.11 (−0.15 to −0.06)	0.90 (0.86 to 0.94)	100	−0.14 (−0.43 to 0.14)	0.87 (0.65 to 1.15)	0.33
ICD10 F60.3; borderline personality disorder	0.04 (0.01 to 0.10)	1.05 (1.01 to 1.11)	93.76	1.15 (0.92 to 1.39)	3.17 (2.50 to 4.02)	<0.001*
ICD F90–F98; behavioural and emotional disorders with onset usually occurring in childhood and adolescence	−0.06 (−0.10 to −0.01)	0.94 (0.90 to 0.99)	99.8	−0.33 (−0.70 to 0.04)	0.72 (0.50 to 1.04)	0.08
ICD F99; unspecified mental disorder	−0.08 (−0.12 to −0.04)	0.92 (0.88 to 0.97)	99.98	0.16 (0.02 to 0.30)	1.18 (1.02 to 1.36)	0.02
ICD 10 O20–O29; other maternal disorders predominantly related to pregnancy	0.06 (0.01 to 0.11)	1.06 (1.01 to 1.11)	99.04	−0.02 (−0.19 to 0.15)	0.98 (0.83 to 1.16)	0.83
ICD10 Z55–Z65; persons with potential health hazards related to socioeconomic and psychosocial circumstances	−0.05 (−0.10 to −0.01)	0.95 (0.90 to 1.00)	98.72	−0.16 (−0.41 to 0.10)	0.86 (0.66 to 1.11)	0.24
ICD10 Z77–Z99; persons with potential health hazards related to family and personal history and certain conditions influencing health status	−0.05 (−0.10 to −0.01)	0.95 (0.90 to 1.00)	98.54	−0.09 (−0.21 to 0.03)	0.91 (0.81 to 1.03)	0.14

The table compares results from the bootstrap elastic net model and the single logistic regression model. For each predictor, the bootstrap elastic net model reports median β coefficients, ORs, 95% CIs and variable incluison probability (VIP), whereas the single logistic regression model reports β coefficients, ORs with 95% CIs and p values. Positive coefficients indicate increased odds of perinatal depression onset, whereas negative coefficients indicate reduced odds.

* P-value significant after false discovery rate (FDR) correction.

† For categorical predictors, repeated single logistic regression estimates indicate an overall effect of the variable as coded in the model, rather than separate level-specific estimates hot-encoded for each specific variable level, as perfomed in elastic-net models.

Models predicting admission to acute psychiatric care or home treatment during perinatal and postnatal were significant, but showed a lower performance (AUC=0.63, table 2). Despite consistently good specificity, reduced sensitivity and F1 scores indicated limited case detection. Among pregnancies associated with depression, a higher probability of acute psychiatric admission or home treatment during the perinatal period was associated with previous antipsychotic use, hypnotic use and Black ethnicity (online supplemental table 5). During the postnatal period, we confirmed associations with antipsychotic use and ethnicity (online supplemental table 6). Results of logistic regressions assessing the effect of each single predictor mostly confirmed the detected effects, although some associations did not survive FDR correction, including age and ICD codes for mental disorders (F10–19, F50–59, F90–98, F99) and for peripartum (O20–29, O85–92) and factors influencing health status and contact with services (Z55–65, Z77-99) (table 3, online supplemental tables 3-6). Models conducted in the downsamples (online supplemental table 7) showed significant performance across all periods and outcomes (p<0.001), with higher performances for F1 and PPV values compared with whole sample models, predictors were also mostly consistent (online supplemental tables 7-13).

## Discussion

In this large pregnancy-level secondary mental healthcare cohort, ~16% of pregnancies were associated with PD, while ~19% of them involved acute psychiatric admission or home treatment in relation to a depressive episode during peripartum, consistent with previous studies in clinically vulnerable populations.^6^ The key finding was that clinical and sociodemographic information collected during the 2 years before conception provided meaningful, although moderate, discrimination of PD onset across pregnancies, with sensitivity and specificity ranging from 61% to 75%, respectively. The predictive performance observed in this study is consistent with previous ML studies using EHR and clinical variables to predict PD, which have generally reported moderate discrimination, with AUCs of approximately 0.70.^10^ By contrast, prediction of acute psychiatric admission or home treatment was weaker, suggesting that preconception information may support early risk stratification but is less suited to capturing severe, state-dependent deterioration.

In this secondary-care cohort, predictors of PD onset mainly reflected pre-existing affective vulnerability and broader psychosocial disadvantage. Greater depressive and positive symptom severity/disorders and previous treatment with antidepressants, antipsychotics or mood stabilisers were detected among significant risk factors, paralleled by history of borderline personality disorder. These findings are consistent with evidence identifying personal history of depression^16^ and severe mental illness, including psychotic and bipolar disorders,^7 17^ as key determinants of PD. They also align with meta-analytic data, showing an increased risk of postnatal relapse among women with bipolar disorder, who are commonly treated with mood stabilisers and antipsychotics,^17^ or in patients with borderline personality disorder.^18^ Among medical conditions, also history of previous pregnancy-related and puerperium-related disorders (eg, early haemorrhage, urinary infections, gestational diabetes) emerged as risk factors, potentially reflecting both biological and psychological pathways related to an increased risk of PD, such as vulnerability to inflammatory and metabolic dysregulation^19 20^ as well as increased health-related anxiety and stress during the subsequent pregnancy.^21^ Younger age and primiparity were also associated with an increased risk of PD onset, in line with epidemiological evidence.^7^ These results possibly reflect a heightened distress or anxiety related to the first transition to parenthood and greater psychosocial vulnerability, in line with a higher risk associated with socioeconomic deprivation^22^ and a lower risk associated with white ethnicity.^23^ An additional finding concerns marital status, with single status associated with lower PD risk and married status with higher risk, contrary to findings from the general population.^24^ In clinical populations, marital status may not adequately reflect the quality of social support, as low relationship satisfaction, partner conflict or violence, and fertility-related or family-related pressures are associated with increased depression and anxiety.^25^ Marital status may, therefore, capture relational stressors rather than protection.

Lower peripartum depression risk was also observed among pregnancies with preconception anxiety, substance-use, childhood-onset or unspecified mental disorders and selected psychosocial or socioeconomic ICD-10 Z codes, despite these factors generally being associated with increased PD risk in population-based studies.^26^ In our secondary-care cohort, these findings may partly reflect differences in service use and diagnostic coding. Women with established psychiatric diagnoses may have received closer perinatal monitoring or ongoing treatment, potentially reducing the risk of incident depressive episodes or reflecting different clinical trajectories. Similarly, ICD Z-codes may capture healthcare engagement as well as adversity. Associations with depressive symptom severity, previous antidepressant and mood stabiliser use, primiparity, marital status, ethnicity and deprivation were broadly consistent across antenatal and postnatal periods. Antenatal depression additionally showed associations with pregnancy-related and metabolic disorders,^19^ whereas postnatal depression was associated with positive symptoms and smoking during pregnancy, consistent with epidemiological evidence.^27^ This association may reflect both biological mechanisms related to stress-response systems and inflammatory pathways, and the role of smoking as a marker of psychosocial adversity, including relationship adjustment and social support, and pre-existing affective vulnerability.

Models predicting perinatal acute psychiatric admission or home treatment among pregnancies associated with depression achieved lower but still significant performance for perinatal and postpartum periods. Significant predictors across both periods reflected prior vulnerability to severe mental illness and greater clinical complexity, including previous antipsychotic and hypnotic use, with the latter potentially reflecting sleep disturbance, which is strongly associated with PD and suicidality.^28^ Predictors also included markers of potential socioeconomic disadvantage, such as Black ethnicity.^23^ This association is consistent with previous literature reporting a higher risk of postnatal psychosis, often associated with depressive episodes, and compulsory psychiatric admission among Black women.^29^ These findings may also reflect structural socioeconomic factors and inequalities in access to and pathways through mental healthcare among minoritised populations.^29^ Finally, although current birth-related variables such as season of delivery, birth with medical or obstetric intervention, and preterm birth were included as predictors, they did not emerge as significant contributors to the models, suggesting that, in this cohort of pregnancies among women with an active referral to mental health services on or before delivery, the obstetric factors may play a less prominent role in predicting perinatal mental health outcomes.

Taken together, these findings suggest that depression onset and acute psychiatric admission during the perinatal period are primarily associated with preconception markers of illness severity, pre-existing psychiatric and medical conditions and socioeconomic factors, with broadly consistent findings across penalised and single regression analyses. However, despite these clinically meaningful associations, routinely collected EHR information alone provided only limited predictive discrimination at the pregnancy level. Especially, acute care can be associated with severe clinical deterioration including onset psychotic or mixed affective features, marked sleep disruption, or acute suicidal or infanticidal risk. Acute psychiatric care needs are dynamic and may evolve during pregnancy or in the early postnatal period. Although static baseline data may help in identifying women at increased risk of depression, they are likely to have limited value in predicting state-dependent clinical deterioration.

Future models may benefit from integrating more proximal and time-varying information, including longitudinal symptoms and dynamic psychosocial and obstetric factors, as broader clinical and psychometric measures have shown higher discrimination.^10^ Biological markers, including inflammatory, hormonal, and metabolic measures, as well as neuroimaging data, may further improve prediction.^20 30^

Several limitations should be acknowledged. The secondary mental healthcare sample limits generalisability to the wider perinatal population. As pregnancy was the unit of analysis and some women contributed more than one pregnancy, within-person correlation was not explicitly accounted for. Missing data were imputed before CV because of computational constraints within the secure NHS/CRIS environment, so some information leakage and optimistic bias cannot be excluded. The primary analyses were also affected by class imbalance. Although downsampled analyses largely confirmed the main findings, the low PPVs suggest that these models may be more useful for risk stratification and closer monitoring than for diagnostic or treatment decisions. The retrospective use of routinely collected EHR data and algorithm-based extraction may also have introduced misclassification and information bias, as diagnoses and symptom measures depend on service use and the completeness of clinical documentation. Future studies should use standardised perinatal measures, validate these findings prospectively and externally, and assess whether the models add value beyond established clinical risk assessment. ML should therefore support, rather than replace, clinical evaluation and validated screening tools. This approach is currently being investigated within the HappyMums Consortium.^9^

In conclusion, routinely collected pre-conception clinical variables can support prediction of PD among women engaged with mental health services. Prior affective illness severity, depressive and psychotic-spectrum features and previous intensive psychiatric treatment identified pregnancies at higher risk. Prediction of acute psychiatric admission or home treatment remained modest, indicating that future models should incorporate longitudinal, time-varying clinical and biological information. These findings support the use of pre-pregnancy data for early risk stratification and closer perinatal monitoring.

## Supplementary material

10.1136/bmjment-2026-302945 online supplemental file 1

## Data Availability

No data are available.
