# Supplementary material for: From preconception risk to perinatal outcomes: machine learning prediction of depression and acute psychiatric care from electronic health records in the UK
Source: BMJ Ment Health. 2026 Nov 10;29(1):e302945. doi: 10.1136/bmjment-2026-302945 (PMC13756378; doi:10.1136/bmjment-2026-302945)
Supplement: Supplementary file 1 [file bmjment-29-1-s001.docx]

**Supplementary Materials**

**From preconception risk to perinatal outcomes: Machine Learning prediction of Depression and acute psychiatric care from Electronic Health Records in the UK**

**METHODS**

- **Supplementary Method 1.** Symptom Extraction and Scale Construction
- **Supplementary Method 2.** ICD-10 Predictors and Pregnancy-Related Variables
- **Supplementary Method 3.** Missing data imputation
- **Supplementary Method 4.** Model Training, Validation, and Predictor Selection

**TABLES**

- **Supplementary Table 1.** Variables included as predictors in the machine learning models.
- **Supplementary Table 2.** Descriptive characteristics of the whole and downsampled (sample size-matched) cohorts stratified by onset of perinatal depressive episodes.
- **Supplementary Table 3.** Bootstrap estimates of predictors associated with the onset of antenatal depression in the whole sample.
- **Supplementary Table 4.** Bootstrap estimates of predictors associated with the onset of postnatal depression in the whole sample.
- **Supplementary Table 5.** Bootstrap estimates of predictors associated with admission to acute psychiatric care or home treatment during the perinatal period in the whole sample.
- **Supplementary Table 6.** Bootstrap estimates of predictors associated with admission to acute psychiatric care or home treatment during the postnatal period in the whole sample.
- **Supplementary Table 7.** Predictive performance of machine learning models for perinatal mental health outcomes across perinatal, antenatal, and postnatal periods in the downsample matched samples.
- **Supplementary Table 8.** Bootstrap estimates of predictors, indexed before conception, associated with the onset of perinatal depression in the downsample matched samples.
- **Supplementary Table 9.** Bootstrap estimates of predictors associated with the onset of antenatal depression in the downsample matched samples.
- **Supplementary Table 10.** Bootstrap estimates of predictors associated with the onset of postnatal depression in the downsample matched samples.
- **Supplementary Table 11.** Bootstrap estimates of predictors associated with admission to acute psychiatric care or home treatment during the perinatal period in the downsample matched samples.
- **Supplementary Table 12.** Bootstrap estimates of predictors associated with admission to acute psychiatric care or home treatment during pregnancy in the downsample matched samples.
- **Supplementary Table 13.** Bootstrap estimates of predictors associated with admission to acute psychiatric care or home treatment during the postnatal period in the downsample matched samples.

**FIGURES**

- **Supplementary Figure 1. Forest plots of predictors of depression onset in the antenatal period.**
- **Supplementary Figure 2. Forest plots of predictors of depression onset in the postnatal period.**

**Supplementary Method 1.**

**Symptom Extraction and Scale Construction**

This approach, based on the CRIS Natural Language Processing (NLP) Applications Library developed by the NIHR Maudsley Biomedical Research Centre, aims to standardize the extraction of symptom data from electronic clinical records. It provides a structured and aggregated framework for analyzing symptomatology, reducing noise arising from highly correlated variables and promoting the use of composite symptom scales rather than individual symptom indicators.

The surveillance methodology applies a binary scoring system, whereby each symptom is assigned a value of 1 if identified as present within a defined observation period. Individual symptoms are then summed to generate the following symptom scales:

- Positive schizophréniform (0–16): Includes positive psychotic symptoms such as delusions, hallucinations, and disorganized thinking or behavior.
- Negative schizophréniform (0–12): Encompasses diminished emotional expression, alogia, avolition, anhedonia, and asociality.
- Depressive (0–21): Captures symptoms related to low mood, including sadness, loss of interest, and hopelessness.
- Manic (0–8): Reflects elevated mood, hyperactivity, and irritability.
- Disorganized (0–8): Includes disorganized speech and behavior.
- Catatonic (0–4): Identifies features such as immobility, staring, mutism, and posturing.

In addition, a separate category of ‘other’ symptoms is retained for features not suitable for aggregation into other scale.

This framework enhances interpretability and usability of NLP-derived symptom data by providing clinically meaningful groupings while maintaining flexibility for research applications. The NLP pipeline automatically derives these scales from routinely recorded clinical text, supporting scalable and reproducible psychiatric research.

**Supplementary Method 2.**

**ICD-10 Predictors and Pregnancy-Related Variables**

The following section reports the full list of ICD-10 diagnostic categories and subcategories included as predictors in the machine learning models, together with pregnancy- and birth-related variables derived from HES and CRIS records. Variables were extracted from the preconception period unless otherwise specified.

Previous medical comorbidities (period 1) were identified using ICD-10 diagnostic codes recorded prior to conception through linkage with HES and CRIS. Diagnoses recorded within CRIS were extracted using validated applications from the CRIS Natural Language Processing (NLP) Library, (Maudsley Biomedical Research Centre. Natural language processing service 2020. <https://www.maudsleybrc.nihr.ac.uk/facilities/clinical-record-interactive-search-cris/natural-language-processing-nlp-service/>). ICD-10 macro-categories (i.e. chapter-level alphabetic codes) were entered as predictors, including A00–B99. C00–D49, D50–D89, E00–E89, G00–G99. H00–H59, H60–H95, I00–I99. J00–J99. K00–K95, L00–L99. M00–M99. N00–N99. P00–P96, Q00–Q99. Second-level ICD-10 subcategories were entered for selected chapters, not included as first level macrocategories. For mental, behavioral and neurodevelopmental disorders, ICD-10 F codes, derived from CRIS, were disaggregated into F01–F09, F10–F19, F20–F29, F40–F48, F50–F59, F60–F69, F70–F79, F80–F89, F90–F98. and F99. For mood disorders, ICD-10 subcodes were grouped into bipolar affective disorders (F30–F31), depressive episodes (F32–F33), and other mood [affective] disorders (F34–F39). For pregnancy-, childbirth- and puerperium-related conditions, ICD-10 O codes were subdivided into O00–O08, O10–O16, O20–O29, O24, O30–O48, O60–O77, O80–O82, O85–O92, and O94–O99. For ICD-10 Z codes, capturing factors influencing health status and contact with health services, macro-categories Z55–Z65, Z69–Z76, and Z77–Z99 were included. In addition, third-level ICD-10 subcodes capturing clinically relevant conditions related to perinatal depression were included, namely obesity (E66.9), mental and behavioral disorders associated with the puerperium (F53), and borderline personality disorder (F60.3). All ICD-10 variables were coded as binary indicators reflecting the presence or absence of a diagnosis prior to conception. For postnatal outcomes, variables extracted for period 2, related to the current pregnancy and birth outcomes, were added as predictors , including smoking during pregnancy (extracted for period 2), season of delivery, birth with medical or obstetric interventions (i.e. episiotomy, cesarean, forceps, anesthetic) and preterm birth. When deliveries at <37 weeks were identified from recorded HES gestational age at birth, or an ICD-10 code from a delivery episode (O601 and O603) pregnancy were coded as preterm, and the conception was not calculated using the extracted weeks.

**Supplementary Method 3.**

**Missing data imputation**

Missing data were imputed at the dataset levels, prior to cross-validation, owing to computational constraints associated with the limited computational capacity available within the secure NHS/CRIS environment. Imputation was performed using a K-Nearest Neighbours (KNN) approach, whereby missing values are estimated as the mean of the N nearest observations (neighbours). Similarity between observations was quantified using Euclidean distance. As N is a free hyperparameter, it was optimized over a grid of 100 linearly spaced candidate values ranging from 10 to 1000. The optimal N was selected by performing imputation on complete cases and computing reconstruction accuracy. Imputed missing values were detected for: age, <0.1%; ethnicity, 19.30%; IMD, 3.30%; marital status, 26.70%.

**Supplementary Method 4.**

**Model Training, Validation, and Predictor Selection**

Models’ evaluation was conducted using a 5-fold nested cross-validation (CV) scheme. Within the inner CV loop, the L_1_–L_2_ penalty mixing parameter was optimized across 10 linearly spaced values between 0 (pure L_2_) and 1 (pure L_1_). The overall penalization strength was additionally optimized using 50 logarithmically spaced values between 10⁻³ and 10². Because nested CV yields a potentially distinct optimal hyperparameter configuration for each outer fold, the final model was defined using the median optimal value of each hyper-parameter across folds. This final model was then retrained and validated in an additional cross-validation loop. Cross-validated predictions were used to compute performance metrics, including accuracy, balanced accuracy (BA), F1 score, sensitivity, specificity, positive predictive value (PPV), negative predictive value (NPV), and area under the receiver operating characteristic curve (AUC). To evaluate whether the model’s performance was significantly better than expected by chance, a permutation test with 5000 iterations was conducted. In each iteration, the outcome labels were randomly permuted to break any true association between predictors and outcome, after which the model was retrained. This procedure yielded a distribution of performance metrics expected under the assumption that no real predictive signal exists in the data (i.e., null distribution). The significance of the observed model performance can be defined as the proportion of permuted models whose performance exceeded that of the true model. We set a significance threshold of *p*<0.005, correcting the threshold for multiple comparisons (*p*<0.05/9). To identify the variables that significantly contributed to prediction, a bootstrap procedure with 5000 resamplings was conducted. At each iteration, the data were resampled with replacement and the model was refit, producing a coefficient distribution for each predictor. From these distributions, bias-corrected and accelerated (BCa) confidence intervals were computed. The BCa method adjusts the percentile-based confidence intervals by correcting for both systematic bias (i.e., whether the bootstrap distribution is shifted relative to the observed estimate) and skewness (i.e., asymmetry in the bootstrap distribution), thereby providing more accurate interval estimates, particularly when coefficient distributions deviate from normality. Predictors were considered to significantly contribute to the model if the BCa confidence interval did not include zero and the median coefficient was non-zero. For significant predictors, we also performed logistic regression assessing the effect of each predictor on the considered outcome, results were corrected. Additionally, the variable inclusion probability (VIP) was calculated for each predictor, defined as the proportion of bootstrap iterations in which the coefficient was non-zero. For analyses conducted on the whole sample, cross-validation folds were stratified to preserve the class distribution, and the model’s loss function was reweighted to penalize the majority class proportionally to its imbalance relative to the minority class. As secondary analyses, given the pronounced class imbalance between pregnancy instances with and without the outcome of interest, analyses were also performed by randomly downsampling the majority class to match the size of the minority class. Downsampling was performed once before performing elastic net analyses.

**Supplementary Table 1.** *Variables included as predictors in the machine learning models. The table reports each predictor variable, its data type, coding scheme or levels, and source of data extraction. Predictors include sociodemographic characteristics, lifestyle factors, reproductive and obstetric variables, previous psychological and pharmacological treatments, psychiatric symptom scores, and medical and psychiatric comorbidities based on ICD-10 diagnostic categories. Data were extracted from the Clinical Record Interactive Search system (CRIS) and linked Hospital Episode Statistics (HES).*

| **Variable** | **Type** | **Levels** | **Data extraction** |
| --- | --- | --- | --- |
| Age | Continuous |  | CRIS |
| Ethnicity | Categorical | 0=White British and other White, 1=Black African and other Black,2=Asian/Mixed/Other | CRIS |
| Marital status | Categorical | 0=Single; 1=Cohabiting; 2= Married 3=Separated/Divorced/Widowed | CRIS |
| Index of Multiple Deprivation | Continuous |  | CRIS |
| Smoking | Binary | 0=No;1=Yes | CRIS |
| Number of Previous pregnancies | Continuous |  | HES |
| Primiparity | Binary | 0=No;1=Yes | HES |
| Season of delivery | Categorical | 1=Winter, 2=Spring, 3=Summer, 4=Autumn | HES |
| Cognitive behavioral therapy | Binary | 0=No;1=Yes | CRIS |
| Family interventions | Binary | 0=No;1=Yes | CRIS |
| Antipsychotics | Binary | 0=No;1=Yes | CRIS |
| Antidepressants | Binary | 0=No;1=Yes | CRIS |
| Mood stabilizers | Binary | 0=No;1=Yes | CRIS |
| Hypnotics | Binary | 0=No;1=Yes | CRIS |
| Preterm birth | Binary | 0=No preterm,; 1=Preterm | HES |
| Birth with medical or obstetric intervention | Binary | 0=Not instrumental;1=Instrumental | HES |
| Depressive symptom score | Counts | Range 0-21, See SM1 method | CRIS |
| Manic symptom score | Counts | Range 0-8, See SM1 method | CRIS |
| Positive symptom score | Counts | Range 0-16, See SM1 method | CRIS |
| Negative symptom score | Counts | Range 0-12, See SM1 method | CRIS |
| Disorganized symptom score | Counts | Range 0-8, See SM1 method | CRIS |
| Catatonic symptom score | Counts | Range 0-4, See SM1 method | CRIS |
| Other symptoms score | Counts | See SM1 method | CRIS |
| ICD10 A00-B99; Certain infectious and parasitic diseases | Binary | 0=No;1=Yes | HES |
| ICD10 C00-D49; Neoplasms | Binary | 0=No;1=Yes | HES |
| ICD10 D50-D89; Diseases of the blood and blood-forming organs and certain disorders involving the immune mechanism | Binary | 0=No;1=Yes | HES |
| ICD10 E00-E89; Endocrine, nutritional and metabolic diseases | Binary | 0=No;1=Yes | HES |
| ICD10 E66.9; Obesity, unspecified | Binary | 0=No;1=Yes | HES |
| ICD10 F01-F09; Mental disorders due to known physiological conditions | Binary | 0=No;1=Yes | CRIS |
| ICD F10-F19; Mental and behavioral disorders due to psychoactive substance use | Binary | 0=No;1=Yes | CRIS |
| ICD F20-F29; Schizophrenia, schizotypal, delusional, and other non-mood psychotic disorders | Binary | 0=No;1=Yes | CRIS |
| ICD 10 F34-39; Other Mood [affective] disorders | Binary | 0=No;1=Yes | CRIS |
| ICD10 F30- F31; Bipolar affective disorder | Binary | 0=No;1=Yes | CRIS |
| ICD 10 F32-F33; Depressive episode | Binary | 0=No;1=Yes | CRIS |
| ICD F40-F48; Anxiety, dissociative, stress-related, somatoform and other nonpsychotic mental disorders | Binary | 0=No;1=Yes | CRIS |
| ICD F50-F59; Behavioral syndromes associated with physiological disturbances and physical factors | Binary | 0=No;1=Yes | CRIS |
| ICD 10 F53; Mental and behavioral disorders associated with the puerperium | Binary | 0=No;1=Yes | CRIS |
| ICD F60-F69; Disorders of adult personality and behavior | Binary | 0=No;1=Yes | CRIS |
| ICD10 F60.3; Borderline personality disorder | Binary | 0=No;1=Yes | CRIS |
| ICD F70-F79; Intellectual disabilities | Binary | 0=No;1=Yes | CRIS |
| ICD F80-F89; Pervasive and specific developmental disorders | Binary | 0=No;1=Yes | CRIS |
| ICD F90-F98; Behavioral and emotional disorders with onset usually occurring in childhood and adolescence | Binary | 0=No;1=Yes | CRIS |
| ICD F99; Unspecified mental disorder | Binary | 0=No;1=Yes | CRIS |
| ICD10 G00-G99; Diseases of the nervous system | Binary | 0=No;1=Yes | HES |
| ICD10 H00-H59; Intraoperative and postprocedural complications and disorders of eye and adnexa, not elsewhere classified | Binary | 0=No;1=Yes | HES |
| ICD10 H60-H95; Diseases of the ear and mastoid process | Binary | 0=No;1=Yes | HES |
| ICD10 I00-I99; Diseases of the circulatory system | Binary | 0=No;1=Yes | HES |
| ICD10 J00-J99; Diseases of the respiratory system | Binary | 0=No;1=Yes | HES |
| ICD10 K00-K95; Diseases of the digestive system | Binary | 0=No;1=Yes | HES |
| ICD10 L00-L99; Diseases of the skin and subcutaneous tissue | Binary | 0=No;1=Yes | HES |
| ICD10 M00-M99; Diseases of the musculoskeletal system and connective tissue | Binary | 0=No;1=Yes | HES |
| ICD10 N00-N99; Diseases of the genitourinary system | Binary | 0=No;1=Yes | HES |
| ICD10 O00-O08; Pregnancy with abortive outcome | Binary | 0=No;1=Yes | HES |
| ICD 10 O10-O16; Edema, proteinuria and hypertensive disorders in pregnancy, childbirth and the puerperium | Binary | 0=No;1=Yes | HES |
| ICD 10 O20-O29; Other maternal disorders predominantly related to pregnancy | Binary | 0=No;1=Yes | HES |
| ICD 10 O24; Diabetes mellitus in pregnancy, childbirth, and the puerperium | Binary | 0=No;1=Yes | HES |
| ICD 10 O30-O48; Maternal care related to the fetus and amniotic cavity and  possible delivery problems | Binary | 0=No;1=Yes | HES |
| ICD 10 O60-O77; Complications of labor and delivery | Binary | 0=No;1=Yes | HES |
| ICD 10 O80-O82; Encounter for delivery | Binary | 0=No;1=Yes | HES |
| ICD 10 O85-O92 Complications predominantly related to the puerperium | Binary | 0=No;1=Yes | HES |
| ICD 10 O94-O99 Other obstetric conditions, not elsewhere classified | Binary | 0=No;1=Yes | HES |
| ICD10 P00-P96; Certain conditions originating in the perinatal period | Binary | 0=No;1=Yes | HES |
| ICD10 Q00-Q99; Congenital malformations, deformations and chromosomal abnormalities | Binary | 0=No;1=Yes | HES |
| ICD10 Z69-Z76; Persons encountering health services in other circumstances | Binary | 0=No;1=Yes | HES |
| ICD10 Z77-Z99; Persons with potential health hazards related to family and personal history and certain conditions influencing health status | Binary | 0=No;1=Yes | HES |
| ICD10 Z55-Z65; Persons with potential health hazards related to socioeconomic and psychosocial circumstances | Binary | 0=No;1=Yes | HES |

**Supplementary Table 2.** Descriptive characteristics of pregnancy instances in the whole and downsampled (sample size-matched) samples, stratified by the occurrence of perinatal depressive episodes. Continuous variables are reported as mean ± standard deviation, and categorical variables as counts and percentages. Pre-conception ICD-10 comorbidities were defined as binary indicators (presence/absence of each diagnosis before conception), including chapter-level categories and selected psychiatric, obstetric, and medically relevant subcategories. Percentages were calculated within groups. Between-group comparisons were performed using independent samples t-tests or χ² tests, as appropriate, and corresponding p-values and effects sizes (Cohen’s d or Cramer’s V) are reported. NE = not estimable due to complete data separation; p < 0.05; ** p significant after false discovery rate (FDR) correction. † **=** postpartum variables included in the postpartum downsampled cohort and subsequently entered into the prediction model.

|  | **Whole sample, n=20793 pregnancies** | | | | | **Downsample, n=6610 pregnancies** | | | | |
| --- | --- | --- | --- | --- | --- | --- | --- | --- | --- | --- |
| **Perinatal Depression** | **Perinatal depression,**  **n=3305** | **No perinatal depression,**  **n=17488** | **t / X2** | **Cohen’s d / Cramer’s V** | ***p*** | **Perinatal depression,  n=3305** | **No perinatal depression,**  **n=3305** | **t / X2** | **Cohen’s d / Cramer’s V** | ***p*** |
| **Preconception indexed variables** | | | | | | | | | | |
| **Age** | 30.83 ± 6.20 | 30.86 ± 6.01 | 0.17 | 0.003 | 0.87 | 30.83 ± 6.20 | 30.81 ± 5.91 | -0.08 |  | 0.94 |
| **Ethnicity**   White british and other White  Black african and other Black  Asian/Mixed/Other | 1669 (50.5%)  1037 (31.4%)  599 (18.1%) | 10661 (61.0%) 4545 (26.0%) 2282 (13.0%) | 133.03 | 0.08 | <0.001** | 1669 (50.5%) 1037 (31.4%) 599 (18.1%) | 1986 (60.1%)  882 (26.7%)  437 (13.2%) | 65.34 | 0.21 | <0.001** |
| **Marital status**  Single   Cohabiting  Married  Separated/divorced/widowed | 1617 (48.9%) 582 (17.6%)  969 (29.3%)  137 (4.2%) | 11721 (67.0%) 2793 (16.0%) 2488 (14.2%)  486 (2.8%) | 544.66 | 0.16 | <0.001** | 1617 (48.9%) 582 (17.6%) 969 (29.3%) 137 (4.2%) | 2248 (68.0%) 506 (15.3%) 451 (13.7%) 100 (3.0%) | 303.06 | 0.21 | <0.001** |
| **Index of multiple Deprivation** | 26.49 ± 9.44 | 25.78 ± 10.47 | 3.59 | 0.07 | <0.001** | 26.49 ± 9.44 | 25.98 ± 10.32 | 2.06 | 0.05 | <0.05* |
| **Number of previous pregnancy** | 1.10 ± 1.39 | 1.35 ± 1.48 | 9.1 | 0.17 | <0.001** | 1.10 ± 1.39 | 1.35 ± 1.48 | 6.98 | 0.17 | <0.001** |
| **Primiparity** | 1452 (43.9%) / 1853 (56.1%) | 5954 (34.0%) / 11534 (66.0%) | 118.07 | 0.08 | <0.001** | 1452 (43.9%) / 1853 (56.1%) | 1138 (34.4% )/ 2167 (65.6%) | 62.2 | 0.10 | <0.001** |
| **Cognitive behavioral therapy / Family interventions** | 119 (3.6%) / 3186 (96.4%) | 275 (1.6%) / 17213 (98.4%) | 60.42 | 0.05 | <0.001** | 119 (3.6%) / 3186 (96.4%) | 61 (1.8%) / 3244 (98.2%) | 18.56 | 0.05 | <0.001** |
| **Use of Antipsychotics** | 460 (13.9%) / 2845 (86.1%) | 520 (3.0%) / 16968 (97.0%) | 738.99 | 0.19 | <0.001** | 460 (13.9%) / 2845 (86.1%) | 87 (2.6%) / 3218 (97.4%) | 275.81 | 0.20 | <0.001** |
| **Use of Antidepressants** | 769 (23.3%) / 2536 (76.7%) | 1434 (8.2%) / 16054 (91.8%) | 664.66 | 0.18 | <0.001** | 769 (23.3%) / 2536 (76.7%) | 256 (7.7%) / 3049 (92.3%) | 302.69 | 0.21 | <0.001** |
| **Use of Mood stabilizers** | 183 (5.5%) / 3122 (94.5%) | 188 (1.1%) / 17300 (98.9%) | 313.27 | 0.12 | <0.001** | 183 (5.5%) / 3122 (94.5%) | 38 (1.1%) / 3267 (98.9%) | 97.07 | 0.12 | <0.001** |
| **Use of Hypnotics** | 395 (12.0%) / 2910 (88.0%) | 557 (3.2%) / 16931 (96.8%) | 486.97 | 0.15 | <0.001** | 395 (12.0%) / 2910 (88.0%) | 95 (2.9%) / 3210 (97.1%) | 197.06 | 0.17 | <0.001** |
| **Depressive symptom score** | 1.84 ± 3.07 | 0.58 ± 1.58 | 34.89 | 0.66 | <0.001** | 1.84 ± 3.07 | 0.54 ± 1.52 | 21.74 | 0.53 | <0.001** |
| **Manic symptom score** | 0.99 ± 1.74 | 0.31 ± 0.87 | 33.63 | 0.64 | <0.001** | 0.99 ± 1.74 | 0.29 ± 0.82 | 20.86 | 0.51 | <0.001** |
| **Positive symptom score** | 1.10 ± 2.39 | 0.27 ± 1.04 | 32.54 | 0.62 | <0.001** | 1.10 ± 2.39 | 0.25 ± 0.97 | 18.97 | 0.47 | <0.001** |
| **Negative symptom score** | 0.44 ± 1.01 | 0.09 ± 0.44 | 31.5 | 0.60 | <0.001** | 0.44 ± 1.01 | 0.10 ± 0.44 | 17.92 | 0.44 | <0.001** |
| **Disorganized symptom score** | 0.33 ± 0.87 | 0.08 ± 0.36 | 27.39 | 0.52 | <0.001** | 0.33 ± 0.87 | 0.07 ± 0.32 | 16.14 | 0.40 | <0.001** |
| **Catatonic symptom score** | 0.03 ± 0.19 | 0.006 ± 0.08 | 13 | 0.25 | <0.001** | 0.03 ± 0.19 | 0.004 ± 0.07 | 8.05 | 0.20 | <0.001** |
| **Other symptoms score** | 0.99 ± 1.49 | 0.39 ± 0.90 | 30.85 | 0.59 | <0.001** | 0.99 ± 1.49 | 0.38 ± 0.88 | 20.25 | 0.50 | <0.001** |
| **ICD10 A00-B99; Certain infectious and parasitic diseases** | 91 (2.8%) /  3214 (97.2%) | 540 (3.1%) /  16948 (96.9%) | 0.95 | 0.01 | 0.33 | 91 (2.8%) /  3214 (97.2%) | 86 (2.6%) /  3219 (97.4%) | 0.09 | <0.01 | 0.76 |
| **ICD10 C00-D49; Neoplasms** | 53 (1.6%) /  3252 (98.4%) | 276 (1.6%) /  17212 (98.4%) | 0.001 | <0.01 | 0.97 | 53 (1.6%) /  3252 (98.4%) | 54 (1.6%) /  3251 (98.4%) | 0 | 0 | 1 |
| **ICD10 D50-D89; Diseases of the blood and blood-forming organs and certain disorders involving the immune mechanism** | 83 (2.5%) /  3222 (97.5%) | 367 (2.1%) /  17121 (97.9%) | 2.05 | 0.01 | 0.15 | 83 (2.5%) /  3222 (97.5%) | 69 (2.1%) /  3236 (97.9%) | 1.14 | 0.01 | 0.29 |
| **ICD10 E00-E89; Endocrine. nutritional and metabolic diseases** | 180 (5.4%) /  3125 (94.6%) | 731 (4.2%) /  16757 (95.8%) | 10.34 | 0.02 | <0.05** | 180 (5.4%) /  3125 (94.6%) | 132 (4.0%) /  3173 (96.0%) | 7.43 | 0.03 | <0.05** |
| **ICD10 E66.9; Obesity. unspecified** | 40 (1.2%) /  3265 (98.8%) | 175 (1.0%) /  17313 (99.0%) | 1 | 0.01 | 0.32 | 40 (1.2%) /  3265 (98.8%) | 29 (0.9%) /  3276 (99.1%) | 1.46 | 0.02 | 0.22 |
| **ICD10 F01-F09; Mental disorders due to known physiological conditions** | 5 (0.2%) /  3300 (99.8%) | 35 (0.2%) /  17453 (99.8%) | 0.14 | <0.01 | 0.71 | 5 (0.2%) /  3300 (99.8%) | 7 (0.2%) /  3298 (99.8%) | 0.08 | <0.01 | 0.77 |
| **ICD F10-F19; Mental and behavioral disorders due to psychoactive substance use** | 74 (2.2%) /  3231 (97.8%) | 365 (2.1%) /  17123 (97.9%) | 0.24 | <0.01 | 0.62 | 74 (2.2%) / 3231 (97.8%) | 88 (2.7%) /  3217 (97.3%) | 1.07 | 0.01 | 0.3 |
| **ICD F20-F29; Schizophrenia. schizotypal. delusional. and other non-mood psychotic disorders** | 132 (4.0%) / 3173 (96.0%) | 99 (0.6%) /  17389 (99.4%) | 294.19 | 0.12 | <0.001** | 132 (4.0%) /  3173 (96.0%) | 17 (0.5%) /  3288 (99.5%) | 89.23 | 0.12 | <0.001** |
| **ICD10 F30- F31; Bipolar affective disorder** | 81 (2.5%) /  3224 (97.5%) | 82 (0.5%) /  17406 (99.5%) | 137.85 | 0.08 | <0.001** | 81 (2.5%) /  3224 (97.5%) | 15 (0.5%) /  3290 (99.5%) | 44.66 | 0.08 | <0.001** |
| **ICD 10 F32-F33; Depressive episode** | 343 (10.4%) / 2962 (89.6%) | 705 (4.0%) /  16783 (96.0%) | 232.63 | 0.09 | <0.001** | 343 (10.4%) /  2962 (89.6%) | 125 (3.8%) /  3180 (96.2%) | 108.28 | 0.13 | <0.001** |
| **ICD 10 F34-39; Other Mood [affective] disorders** | 30 (0.9%) /  3275 (99.1%) | 92 (0.5%) /  17396 (99.5%) | 6.3 | 0.02 | <0.05** | 30 (0.9%) /  3275 (99.1%) | 17 (0.5%) /  3288 (99.5%) | 3.09 | 0.02 | 0.08 |
| **ICD F40-F48; Anxiety. dissociative. stress-related. somatoform and other nonpsychotic mental disorders** | 178 (5.4%) /  3127 (94.6%) | 741 (4.2%) /  16747 (95.8%) | 8.41 | 0.02 | <0.05** | 178 (5.4%) /  3127 (94.6%) | 149 (4.5%) /  3156 (95.5%) | 2.52 | 0.02 | 0.11 |
| **ICD F50-F59; Behavioral syndromes associated with physiological disturbances and physical factors** | 56 (1.7%) /  3249 (98.3%) | 341 (1.9%) /  17147 (98.1%) | 0.84 | 0.01 | 0.36 | 56 (1.7%) /  3249 (98.3%) | 74 (2.2%) /  3231 (97.8%) | 2.27 | 0.02 | 0.13 |
| **ICD 10 F53;Mental and behavioral disorders associated with the puerperium** | 11 (0.3%) /  3294 (99.8%) | 23 (0.1%) /  17465 (99.9%) | 5.72 | 0.02 | <0.05** | 11 (0.3%) /  3294 (99.8%) | 7 (0.2%) /  3298 (99.8%) | 0.50 | 0.01 | 0.48 |
| **ICD F60-F69; Disorders of adult personality and behavior** | 129 (3.9%) /  3176 (96.1%) | 250 (1.4%) /  17238 (98.6%) | 93.67 | 0.07 | <0.001** | 129 (3.9%) /  3176 (96.1%) | 53 (1.6%) /  3252 (98.4%) | 31.78 | 0.07 | <0.001** |
| **ICD10 F60.3; Borderline personality disorder** | 110 (3.3%) /  3195 (96.7%) | 188 (1.1%) /  17300 (98.9%) | 98.32 | 0.07 | <0.001** | 110 (3.3%) /  3195 (96.7%) | 40 (1.2%) /  3265 (98.8%) | 32.48 | 0.07 | <0.001** |
| **ICD F70-F79; Intellectual disabilities** | 6 (0.2%) /  3299 (99.8%) | 17 (<0.1%) / 17471 (~100%) | 1.11 | 0.01 | 0.29 | 6 (0.2%) /  3299 (99.8%) | 1 (<0.1%) /  3304 (~100%) | 2.28 | 0.02 | 0.13 |
| **ICD F80-F89; Pervasive and specific developmental disorders** | 7 (0.2%) /  3298 (99.8%) | 34 (0.2%) / 17454 (99.8%) | 0 | 0 | 1 | 7 (0.2%) /  3298 (99.8%) | 8 (0.2%) /  3297 (99.8%) | 0 | 0 | 1.00 |
| **ICD F90-F98; Behavioral and emotional disorders with onset usually occurring in childhood and adolescence** | 32 (1.0%) /  3273 (99.0%) | 234 (1.3%) / 17254 (98.7%) | 2.72 | 0.01 | 0.1 | 32 (1.0%) /  3273 (99.0%) | 37 (1.1%) /  3268 (98.9%) | 0.23 | 0.01 | 0.63 |
| **ICD F99; Unspecified mental disorder** | 251 (7.6%) / 3054 (92.4%) | 1142 (6.5%) /  16346 (93.5%) | 4.87 | 0.02 | <0.05* | 251 (7.6%) /  3054 (92.4%) | 205 (6.2%) /  3100 (93.8%) | 4.77 | 0.03 | <0.05* |
| **ICD10 G00-G99; Diseases of the nervous system** | 88 (2.7%) /  3217 (97.3%) | 462 (2.6%) /  17026 (97.4%) | 0 | <0.01 | 1 | 88 (2.7%) /  3217 (97.3%) | 99 (3.0%) /  3206 (97.0%) | 0.55 | 0.01 | 0.46 |
| **ICD10 H00-H59; Intraoperative and postprocedural complications and disorders of eye and adnexa. not elsewhere classified** | 25 (0.8%) /  3280 (99.2%) | 179 (1.0%) /  17309 (99.0%) | 1.77 | 0.01 | 0.18 | 25 (0.8%) /  3280 (99.2%) | 31 (0.9%) /  3274 (99.1%) | 0.45 | 0.01 | 0.5 |
| **ICD10 H60-H95; Diseases of the ear and mastoid process** | 15 (0.5%) /  3290 (99.5%) | 50 (0.3%) /  17438 (99.7%) | 2 | 0.01 | 0.16 | 15 (0.5%) /  3290 (99.5%) | 4 (0.1%) /  3301 (99.9%) | 5.28 | 0.03 | <0.05* |
| **ICD10 I00-I99; Diseases of the circulatory system** | 75 (2.3%) /  3230 (97.7%) | 367 (2.1%) /  17121 (97.9%) | 0.31 | <0.01 | 0.58 | 75 (2.3%) /  3230 (97.7%) | 78 (2.4%) /  3227 (97.6%) | 0.03 | <0.01 | 0.87 |
| **ICD10 J00-J99; Diseases of the respiratory system** | 208 (6.3%) /  3097 (93.7%) | 1287 (7.4%) /  16201 (92.6%) | 4.57 | 0.01 | <0.05* | 208 (6.3%) /  3097 (93.7%) | 248 (7.5%) /  3057 (92.5%) | 3.58 | 0.02 | 0.06 |
| **ICD10 K00-K95; Diseases of the digestive system** | 298 (9.0%) /  3007 (91.0%) | 1480 (8.5%) /  16008 (91.5%) | 1.02 | 0.01 | 0.31 | 298 (9.0%) /  3007 (91.0%) | 273 (8.3%) /  3032 (91.7%) | 1.1 | 0.012 | 0.29 |
| **ICD10 L00-L99; Diseases of the skin and subcutaneous tissue** | 60 (1.8%) /  3245 (98.2%) | 308 (1.8%) /  17180 (98.2%) | 0.02 | <0.01 | 0.88 | 60 (1.8%) /  3245 (98.2%) | 59 (1.8%) /  3246 (98.2%) | 0 | 0 | 1.00 |
| **ICD10 M00-M99; Diseases of the musculoskeletal system and connective tissue** | 144 (4.4%) /  3161 (95.6%) | 753 (4.3%) /  16735 (95.7%) | 0.01 | <0.01 | 0.93 | 144 (4.4%) /  3161 (95.6%) | 144 (4.4%) /  3161 (95.6%) | 0 | 0 | 1.00 |
| **ICD10 N00-N99; Diseases of the genitourinary system** | 255 (7.7%) /  3050 (92.3%) | 1370 (7.8%) /  16118 (92.2%) | 0.04 | <0.01 | 0.84 | 255 (7.7%) /  3050 (92.3%) | 232 (7.0%) /  3073 (93.0%) | 1.07 | 0.01 | 0.3 |
| **ICD10 O00-O08; Pregnancy with abortive outcome** | 250 (7.6%) /  3055 (92.4%) | 1381 (7.9%) /  16107 (92.1%) | 0.38 | <0.01 | 0.54 | 250 (7.6%) /  3055 (92.4%) | 271 (8.2%) /  3034 (91.8%) | 0.83 | 0.01 | 0.36 |
| **ICD 10 O10-O16; Edema. proteinuria and hypertensive disorders in pregnancy. childbirth and the puerperium** | 33 (1.0%) /  3272 (99.0%) | 163 (0.9%) /  17325 (99.1%) | 0.07 | <0.01 | 0.79 | 33 (1.0%) /  3272 (99.0%) | 26 (0.8%) /  3279 (99.2%) | 0.62 | 0.01 | 0.43 |
| **ICD 10 O20-O29; Other maternal disorders predominantly related to pregnancy** | 171 (5.2%) /  3134 (94.8%) | 921 (5.3%) /  16567 (94.7%) | 0.03 | <0.01 | 0.86 | 171 (5.2%) /  3134 (94.8%) | 158 (4.8%) /  3147 (95.2%) | 0.46 | 0.01 | 0.5 |
| **ICD 10 O24; Diabetes mellitus in pregnancy. childbirth. and the puerperium** | 9 (0.3%) /  3296 (99.7%) | 49 (0.3%) /  17439 (99.7%) | 0 | 0 | 1 | 9 (0.3%) /  3296 (99.7%) | 11 (0.3%) /  3294 (99.7%) | 0.05 | <0.01 | 0.82 |
| **ICD 10 O30-O48; Maternal care related to the fetus and amniotic cavity and possible delivery problems** | 146 (4.4%) /  3159 (95.6%) | 943 (5.4%) /  16545 (94.6%) | 5.12 | 0.02 | <0.05* | 146 (4.4%) /  3159 (95.6%) | 165 (5.0%) /  3140 (95.0%) | 1.09 | <0.01 | 0.3 |
| **ICD 10 O60-O77; Complications of labor and delivery** | 166 (5.0%) / 3139 (95.0%) | 1091 (6.2%) /  16397 (93.8%) | 7.02 | 0.02 | <0.05** | 166 (5.0%) /  3139 (95.0%) | 196 (5.9%) /  3109 (94.1%) | 2.46 | 0.02 | 0.12 |
| **ICD 10 O80-O82; Encounter for delivery** | 42 (1.3%) /  3263 (98.7%) | 288 (1.6%) /  17200 (98.4%) | 2.28 | 0.01 | 0.13 | 42 (1.3%) /  3263 (98.7%) | 54 (1.6%) /  3251 (98.4%) | 1.28 | 0.01 | 0.26 |
| **ICD 10 O85-O92 Complications predominantly related to the puerperium** | 13 (0.4%) /  3292 (99.6%) | 72 (0.4%) /  17416 (99.6%) | 0 | <0.01 | 1 | 13 (0.4%) /  3292 (99.6%) | 9 (0.3%) /  3296 (99.7%) | 0.41 | 0.01 | 0.52 |
| **ICD 10 O94-O99 Other obstetric conditions. not elsewhere classified** | 49 (1.5%) /  3256 (98.5%) | 305 (1.7%) /  17183 (98.3%) | 0.98 | 0.01 | 0.32 | 49 (1.5%) /  3256 (98.5%) | 53 (1.6%) /  3252 (98.4%) | 0.09 | <0.01 | 0.76 |
| **ICD10 P00-P96; Certain conditions originating in the perinatal period** | 0 (0%) /  3305 (100%) | 1 (<0.1%) /  17487 (~100%) | NE | NE | NE | 0 (0%) /  3305 (100%) | 0 (0%) /  3305 (100.0%) | NE | NE | NE |
| **ICD10 Q00-Q99; Congenital malformations. deformations and chromosomal abnormalities** | 14 (0.4%) /  3291 (99.6%) | 123 (0.7%) /  17365 (99.3%) | 2.91 | 0.01 | 0.09 | 14 (0.4%) /  3291 (99.6%) | 31 (0.9%) /  3274 (99.1%) | 5.73 | 0.03 | <0.05* |
| **ICD10 Z55-Z65; Persons with potential health hazards related to socioeconomic and psychosocial circumstances** | 68 (2.1%) /  3237 (97.9%) | 419 (2.4%) /  17069 (97.6%) | 1.25 | 0.01 | 0.26 | 68 (2.1%) /  3237 (97.9%) | 68 (2.1%) /  3237 (97.9%) | 0 | 0 | 1.00 |
| **ICD10 Z69-Z76; Persons encountering health services in other circumstances** | 70 (2.1%) /  3235 (97.9%) | 520 (3.0%) /  16968 (97.0%) | 7.07 | 0.02 | <0.05** | 70 (2.1%) /  3235 (97.9%) | 91 (2.8%) /  3214 (97.2%) | 2.55 | 0.02 | 0.11 |
| **ICD10 Z77-Z99; Persons with potential health hazards related to family and personal history and certain conditions influencing health status** | 345 (10.4%) /  2960 (89.6%) | 1982 (11.3%) /  15506 (88.7%) | 2.15 | 0.01 | 0.14 | 345 (10.4%) /  2960 (89.6%) | 384 (11.6%) /  2921 (88.4%) | 2.23 | 0.02 | 0.14 |
| **Perinatal indexed variables** | | | | | | | | | | |
| **Season of delivery**†  Spring   Summer  Autumn  Winter | 858 (26.0%)  843 (25.5%)  823 (24.9%)  781 (23.6%) | 4499 (25.7%) 4400 (25.2%) 4338 (24.8%) 4251 (24.3%) | 0.73 | 0.01 | 0.87 | 576 (26.2%) 544 (24.8%) 541 (24.6%) 536 (24.4%) | 568 (25.8%)  587 (26.7%)  522 (23.8%)  520 (23.7%) | 2.27 | 0.02 | 0.52 |
| **Smoking during pregnancy** | 373 (11.3%) / 2932 (88.7%) | 925 (5.3%) / 16563 (94.7%) | 169.76 | 0.09 | <0.001** | 373 (11.3%) / 2932 (88.7%) | 158 (4.8%) / 3147 (95.2%) | 93.78 | 0.21 | <0.001** |
| **Preterm birth** † | 411 (12.4%) / 2894 (87.6%) | 2140 (12.2%) / 15348 (87.8%) | 0.084 | <0.01 | 0.77 | 296 (13.5%) / 1901 (86.5%) | 271 (12.3%) / 1926 (87.7%) | 1.17 | 0.02 | 0.28 |
| **Birth with medical or obstetric intervention** † | 2403 (72.7%) / 902 (27.3%) | 12814 (73.3%) / 4674 (26.7%) | 0.42 | 0.01 | 0.51 | 1594 (72.6%) / 603 (27.4%) | 1589 (72.3%) / 608 (27.7%) | 0.02 | 0.02 | 0.89 |

**Supplementary Table 3.** Bootstrap estimates of predictors associated with the onset of antenatal depression in the whole samples. The table compares results from the bootstrap elastic net model and the single logistic regression model. For each predictor, the bootstrap elastic net model reports median β coefficients, odds ratios (ORs), 95% confidence intervals (CIs), and variable importance in projection (VIP), whereas the single logistic regression model reports β coefficients, ORs with 95% CIs, and p-values. Positive coefficients indicate increased odds of antenatal depression onset, whereas negative coefficients indicate reduced odds. † For categorical predictors, repeated single logistic regression estimates indicate an overall effect of the variable as coded in the model, rather than separate level-specific estimates. NE = not estimable because of complete data separation. * = p-value significant; ** = p-value significant after false discovery rate (FDR) correction.

|  | **Bootstrap performance elastic net model** | | | **Bootstrap performance single logistic regression** | | |
| --- | --- | --- | --- | --- | --- | --- |
| **Predictors** | **Median β (95% CI)** | **Odds ratio (95% CI)** | **VIP** | **β (95% CI)** | **Odds Ratio (95% CI)** | **p** |
| **White ethnicity (vs other ethnic groups)†** | -0.14 (-0.20 - -0.08) | 0.87 (0.82 - 0.93) | 99.98 | 0.28 (0.22 - 0.35) | 1.33 (1.25 - 1.41) | <0.001** |
| **Single marital status (vs cohabiting/married)†** | -0.28 (-0.35 - -0.23) | 0.76 (0.70 - 0.80) | 100 | 0.43 (0.38 - 0.48) | 1.53 (1.46 - 1.61) | <0.001** |
| **Married status (vs single/cohabiting)†** | 0.24 (0.17 - 0.28) | 1.27 (1.18 - 1.32) | 100 |  |  |  |
| **Index of Multiple Deprivation** | 0.10 (0.04 - 0.15) | 1.11 (1.04 - 1.16) | 100 | 0.01 (0.00 - 0.01) | 1.01 (1.00 - 1.01) | <0.05** |
| **Number of Previous pregnancies** | -0.11 (-0.18 - -0.02) | 0.90 (0.83 - 0.98) | 99.46 | -0.13 (-0.17 - -0.10) | 0.88 (0.85 - 0.91) | <0.001** |
| **Primiparity (first pregnancy)** | 0.21 (0.13 - 0.28) | 1.23 (1.14 - 1.32) | 100 | 0.38 (0.28 - 0.48) | 1.46 (1.33 - 1.61) | <0.001** |
| **Use of antidepressants** | 0.19 (0.13 - 0.25) | 1.21 (1.14 - 1.28) | 100 | 1.42 (1.30 - 1.53) | 4.13 (3.68 - 4.63) | <0.001** |
| **Use of mood stabilizers** | 0.09 (0.05 - 0.13) | 1.10 (1.05 - 1.14) | 100 | 1.81 (1.57 - 2.04) | 6.10 (4.82 - 7.71) | <0.001** |
| **Depressive symptom severity** | 0.26 (0.16 - 0.38) | 1.30 (1.17 - 1.46) | 100 | 0.27 (0.25 - 0.29) | 1.31 (1.28 - 1.33) | <0.001** |
| **ICD10 E00-E89; Endocrine, nutritional and metabolic diseases** | 0.06 (0.00 - 0.12) | 1.06 (1.00 - 1.12) | 97.68 | 0.37 (0.17 - 0.58) | 1.45 (1.18 - 1.78) | <0.001** |
| **ICD F10-F19; Mental and behavioral disorders due to psychoactive substance use** | -0.08 (-0.13 - -0.01) | 0.92 (0.88 - 0.99) | 99.70 | 0.10 (-0.22 - 0.41) | 1.10 (0.80 - 1.51) | 0.54 |
| **ICD F20-F29; Schizophrenia, schizotypal, delusional, and other non-mood psychotic disorders** | 0.05 (0.01 - 0.10) | 1.05 (1.01 - 1.11) | 99 | 2.04 (1.74 - 2.33) | 7.66 (5.68 - 10.32) | <0.001** |
| **ICD 10 F34-39; Other Mood [affective] disorders** | -0.05 (-0.09 - -0.01) | 0.95 (0.92 - 0.99) | 99.40 | 0.64 (0.14 - 1.14) | 1.90 (1.15 - 3.11) | <0.05** |
| **ICD F40-F48; Anxiety, dissociative, stress-related, somatoform and other nonpsychotic mental disorders** | -0.12 (-0.17 - -0.06) | 0.89 (0.85 - 0.95) | 100 | 0.32 (0.11 - 0.53) | 1.38 (1.12 - 1.69) | <0.05** |
| **ICD-10 F50-F59: Behavioral syndromes associated with physiological disturbances** | -0.12 (-0.17 - -0.05) | 0.89 (0.85 - 0.95) | 100 | 0.04 (-0.29 - 0.38) | 1.04 (0.75 - 1.46) | 0.8 |
| **ICD F90-F98; Behavioral and emotional disorders with onset usually occurring in childhood and adolescence** | -0.07 (-0.13 - -0.01) | 0.93 (0.88 - 0.99) | 99.84 | -0.41 (-0.91 - 0.08) | 0.66 (0.40 - 1.08) | 0.1 |
| **ICD F99; Unspecified mental disorder** | -0.10 (-0.15 - -0.04) | 0.91 (0.86 - 0.96) | 99.98 | 0.20 (0.03 - 0.38) | 1.22 (1.03 - 1.46) | <0.05** |
| **ICD 10 O20-O29; Other maternal disorders predominantly related to pregnancy** | 0.07 (0.01 - 0.14) | 1.07 (1.01 - 1.15) | 98.92 | 0.02 (-0.19 - 0.23) | 1.02 (0.83 - 1.26) | 0.84 |
| **ICD 10 O60-O77; Complications of labor and delivery** | -0.08 (-0.15 - -0.01) | 0.92 (0.86 - 0.99) | 99.42 | -0.30 (-0.52 - -0.08) | 0.74 (0.59 - 0.92) | <0.05** |
| **ICD 10 O85-O92 Complications predominantly related to the puerperium** | 0.07 (0.01 - 0.12) | 1.07 (1.01 - 1.13) | 98.50 | 0.24 (-0.42 - 0.90) | 1.27 (0.65 - 2.46) | 0.48 |
| **ICD10 Z77-Z99; Persons with potential health hazards related to family and personal history and certain conditions influencing health status** | -0.09 (-0.15 - -0.02) | 0.92 (0.86 - 0.98) | 99.72 | -0.11 (-0.26 - 0.05) | 0.90 (0.77 - 1.05) | 0.17 |

**Supplementary Table 4.** Bootstrap estimates of predictors associated with the onset of postnatal depression in the whole sample. Except for smoking during pregnancy, all predictors are indexed before pregnancy. The table compares results from the bootstrap elastic net model and the single logistic regression model. For each predictor, the bootstrap elastic net model reports median β coefficients, odds ratios (ORs), 95% confidence intervals (CIs), and variable importance in projection (VIP), whereas the single logistic regression model reports β coefficients, ORs with 95% CIs, and p-values. Positive coefficients indicate increased odds of postnatal depression onset, whereas negative coefficients indicate reduced odds. † For categorical predictors, repeated single logistic regression estimates indicate an overall effect of the variable as coded in the model, rather than separate level-specific estimates. * = p-value significant; ** = p-value significant after false discovery rate (FDR) correction.

|  | **Bootstrap performance elastic net model** | | | **Bootstrap performance single logistic regression** | | |
| --- | --- | --- | --- | --- | --- | --- |
| **Predictors** | **Median β (95% CI)** | **Odds ratio (95% CI)** | **VIP** | **β (95% CI)** | **Odds Ratio (95% CI)** | **p** |
| **White ethnicity (vs other ethnic groups)** | -0.16 (-0.23 - -0.13) | 0.85 (0.80 - 0.88) | 100 | 0.31 (0.25 - 0.37) | 1.36 (1.28 - 1.44) | <0.001** |
| **Single marital status (vs cohabiting/married)†** | -0.30 (-0.36 - -0.25) | 0.74 (0.70 - 0.78) | 100 | 0.43 (0.39 - 0.48) | 1.54 (1.48 - 1.62) | <0.001** |
| **Married status (vs single/cohabiting)†** | 0.20 (0.14 - 0.24) | 1.22 (1.15 - 1.27) | 100 |  |  |  |
| **Index of Multiple Deprivation** | 0.09 (0.05 - 0.14) | 1.10 (1.05 - 1.15) | 100 | 0.01 (0.00 - 0.01) | 1.01 (1.00 - 1.01) | <0.001** |
| **Primiparity (first pregnancy)** | 0.16 (0.10 - 0.21) | 1.17 (1.10 - 1.24) | 100 | 0.36 (0.27 - 0.45) | 1.43 (1.31 - 1.57) | <0.001** |
| **Smoking during pregnancy** | 0.29 (0.25 - 0.33) | 1.34 (1.29 - 1.39) | 100 | 1.71 (1.57 - 1.85) | 5.53 (4.79 - 6.39) | <0.001** |
| **Use of antidepressants** | 0.12 (0.07 - 0.17) | 1.12 (1.07 - 1.18) | 99.98 | 1.25 (1.14 - 1.37) | 3.51 (3.13 - 3.92) | <0.001** |
| **Use of antipsychotics** | 0.06 (0.01 - 0.11) | 1.06 (1.01 - 1.11) | 98.64 | 1.80 (1.66 - 1.95) | 6.06 (5.24 - 7.00) | <0.001** |
| **Use of mood stabilizers** | 0.08 (0.03 - 0.11) | 1.08 (1.03 - 1.12) | 99.96 | 1.80 (1.57 - 2.02) | 6.03 (4.81 - 7.55) | <0.001** |
| **Depressive symptom severity** | 0.14 (0.06 - 0.21) | 1.15 (1.06 - 1.24) | 99.98 | 0.25 (0.23 - 0.27) | 1.28 (1.26 - 1.31) | <0.001** |
| **Positive symptom score** | 0.07 (0.00 - 0.13) | 1.07 (1.00 - 1.14) | 98.08 | 0.32 (0.29 - 0.34) | 1.38 (1.34 - 1.41) | <0.001** |
| **ICD F10-F19; Mental and behavioral disorders due to psychoactive substance use** | -0.07 (-0.12 - -0.02) | 0.93 (0.89 - 0.98) | 99.92 | 0.13 (-0.17 - 0.42) | 1.14 (0.85 - 1.53) | 0.39 |
| **ICD-10 F40-F48: Anxiety and stress-related disorders** | -0.05 (-0.09 - 0.00) | 0.95 (0.91 - 1.00) | 98.30 | 0.34 (0.14 - 0.53) | 1.40 (1.15 - 1.70) | <0.001** |
| **ICD-10 F50-F59: Behavioral syndromes associated with physiological disturbances** | -0.07 (-0.11 - -0.01) | 0.93 (0.89 - 0.98) | 99.94 | -0.10 (-0.43 - 0.24) | 0.91 (0.65 - 1.27) | 0.58 |
| **ICD10 Z69-Z76; Persons encountering health services in other circumstances** | -0.07 (-0.13 - -0.02) | 0.93 (0.88 - 0.98) | 99.68 | -0.50 (-0.83 - -0.18) | 0.60 (0.44 - 0.84) | <0.05** |

**Supplementary Table 5.** Bootstrap estimates of predictors associated with admission to acute psychiatric care or home treatment during the perinatal period in the whole sample. The table compares results from the bootstrap elastic net model and the single logistic regression model. For each predictor, the bootstrap elastic net model reports median β coefficients, odds ratios (ORs), 95% confidence intervals (CIs), and variable importance in projection (VIP), whereas the single logistic regression model reports β coefficients, ORs with 95% CIs, and p-values. Positive coefficients indicate increased odds of admission to acute psychiatric care or home treatment during the perinatal period. † For categorical predictors, repeated single logistic regression estimates indicate an overall effect of the variable as coded in the model, rather than separate level-specific estimates. * = p-value significant; ** = p-value significant after false discovery rate (FDR) correction.

|  | **Bootstrap performance elastic net model** | | | **Bootstrap performance single logistic regression** | | |
| --- | --- | --- | --- | --- | --- | --- |
| **Predictors** | **Median β (95% CI)** | **Odds ratio (95% CI)** | **VIP** | **β (95% CI)** | **Odds Ratio (95% CI)** | **p** |
| **Black African and other Black ethnicity (vs White/other)†** | 0.15 (0.04 - 0.26) | 1.16 (1.04 - 1.30) | 99.44 | 0.20 (0.08 - 0.31) | 1.22 (1.10 - 1.36) | <0.001** |
| **Use of antipsychotics** | 0.31 (0.23 - 0.43) | 1.36 (1.26 - 1.53) | 100 | 1.65 (1.44 - 1.86) | 5.21 (4.22 - 6.44) | <0.001** |
| **Use of hypnotics** | 0.13 (0.03 – 0.24) | 1.14 (1.03 - 1.27) | 99.14 | 1.45 (1.23 - 1.68) | 4.28 (3.42 - 5.35) | <0.001** |

**Supplementary Table 6.** Bootstrap estimates of predictors associated with admission to acute psychiatric care or home treatment during the postnatal period in the whole sample. The table compares results from the bootstrap elastic net model and the single logistic regression model. For each predictor, the bootstrap elastic net model reports median β coefficients, odds ratios (ORs), 95% confidence intervals (CIs), and variable importance in projection (VIP), whereas the single logistic regression model reports β coefficients, ORs with 95% CIs, and p-values. Positive coefficients indicate increased odds of admission to acute psychiatric care or home treatment during the postnatal period. † For categorical predictors, repeated single logistic regression estimates indicate an overall effect of the variable as coded in the model, rather than separate level-specific estimates. * = p-value significant; ** = p-value significant after false discovery rate (FDR) correction.

|  | **Bootstrap performance elastic net model** | | | **Bootstrap performance single logistic regression** | | |
| --- | --- | --- | --- | --- | --- | --- |
| **Predictors** | **Median β (95% CI)** | **Odds ratio (95% CI)** | **VIP** | **β (95% CI)** | **Odds Ratio (95% CI)** | **p** |
| **Black African and other Black ethnicity (vs White/other)†** | 0.15 (0.04 - 0.29) | 1.16 (1.04 - 1.33) | 98.4 | 0.17 (0.04 - 0.31) | 1.19 (1.04 - 1.36) | <0.05* |
| **Use of antipsychotics** | 0.41 (0.29 - 0.54) | 1.50 (1.33 - 1.71) | 100 | 1.71 (1.46 - 1.96) | 5.53 (4.31 - 7.09) | <0.001** |

**Supplementary Table 7.** Predictive performance of machine learning models for perinatal mental health outcomes across perinatal, antenatal, and postnatal periods in the downsamples. Performance metrics include area under the receiver operating characteristic curve (AUC), accuracy, balanced accuracy (BA), F1 score, negative predictive value (NPV), positive predictive value (PPV), sensitivity, and specificity. NS, Not significant.

|  | **AUC** | **Accuracy** | **BA** | **F1** | **Sensitivity** | **Specificity** | **NPV** | **PPV** | **p-value** |
| --- | --- | --- | --- | --- | --- | --- | --- | --- | --- |
| **Onset of depressive episodes** | | | | | | | | | |
| Perinatal | 0.68 | 67.68 | 67.68 | 65.68 | 61.85 | 73.53 | 65.84 | 70.02 | <0.001 |
| Antenatal | 0.68 | 68.33 | 68.33 | 66.46 | 62.77 | 73.89 | 66.49 | 70.62 | <0.001 |
| Postnatal | 0.69 | 68.89 | 68.89 | 67.52 | 64.68 | 73.10 | 67.42 | 70.63 | <0.001 |
| **Admission to acute psychiatric care or home treatment** | | | | | | | | | |
| Perinatal | 0.63 | 63.37 | 63.37 | 51.54 | 38.97 | 87.76 | 58.98 | 76.10 | <0.001 |
| Antenatal | 0.63 | 63.45 | 63.45 | 53.30 | 41.70 | 85.20 | 59.38 | 73.81 | <0.001 |
| Postnatal | 0.63 | 62.58 | 62.58 | 54.92 | 45.59 | 79.57 | 59.39 | 69.06 | <0.001 |

**Supplementary Table 8.** Bootstrap estimates of predictors, indexed before conception, associated with the onset of perinatal depression in the downsampled cohort. The table compares results from the bootstrap elastic net model and the single logistic regression model. For each predictor, the bootstrap elastic net model reports median β coefficients, odds ratios (ORs), 95% confidence intervals (CIs), and variable importance in projection (VIP), whereas the single logistic regression model reports β coefficients, ORs with 95% CIs, and p-values. Positive coefficients indicate increased odds of perinatal depression onset, whereas negative coefficients indicate reduced odds. † For categorical predictors, repeated single logistic regression estimates indicate an overall effect of the variable as coded in the model, rather than separate level-specific estimates. * = p-value significant; ** = p-value significant after false discovery rate (FDR) correction.

|  | **Bootstrap performance elastic net model** | | | **Bootstrap performance single logistic regression** | | |
| --- | --- | --- | --- | --- | --- | --- |
| **Predictors** | **Median β (95% CI)** | **Odds ratio (95% CI)** | **VIP** | **β (95% CI)** | **Odds Ratio (95% CI)** | ***p*** |
| **White ethnicity (vs other ethnic groups)†** | -0.12 (-0.19  - -0.08) | 0.89 (0.83  - 0.92) | 100 | 0.26 (0.20  - 0.33) | 1.30 (1.22  - 1.39) | <0.001 |
| **Single marital status (vs cohabiting/married)†** | -0.31 (-0.38  - -0.25) | 0.73 (0.69  - 0.78) | 100 | 0.45 (0.39  - 0.50) | 1.56 (1.48  - 1.65) | <0.001 |
| **Married status (vs single/cohabiting)†** | 0.22 (0.15  - 0.28) | 1.24 (1.16  - 1.32) | 100 |  |  |  |
| **Index of Multiple Deprivation** | 0.06 (0.01  - 0.11) | 1.08 (1.01  - 1.12) | 98.7 | 0.01 (0.001  - 0.01) | 1.01 (1.00  - 1.01) | <0.05 |
| **Primiparity (first pregnancy)** | 0.19 (0.12  - 0.26) | 1.21 (1.13  - 1.29) | 100 | 0.40 (0.30  - 0.50) | 1.49 (1.35  - 1.65) | <0.001 |
| **Use of  antidepressants** | 0.18 (0.11  - 0.26) | 1.20 (1.11  - 1.30) | 100 | 1.28 (1.13  - 1.44) | 3.61 (3.11  - 4.20) | <0.001 |
| **Use of antipsychotics** | 0.08 (0.00  - 0.18) | 1.09 (1.00  - 1.19) | 96.68 | 1.79 (1.55  - 2.02) | 5.98 (4.73  - 7.56) | <0.001 |
| **Use of mood stabilizers** | 0.08 (0.01  - 0.15) | 1.08 (1.01  - 1.16) | 98.88 | 1.62 (1.26  - 1.97) | 5.04 (3.54  - 7.17) | <0.001 |
| **Depressive symptom severity** | 0.29 (0.20  - 0.46) | 1.34 (1.22  - 1.58) | 100 | 0.26 (0.23  - 0.29) | 1.29 (1.26  - 1.33) | <0.001 |
| **Positive symptom severity** | 0.10 (0.01  - 0.26) | 1.11 (1.01  - 1.30) | 94.48 | 0.35 (0.30  - 0.39) | 1.41 (1.35  - 1.48) | <0.001 |
| **ICD-10 F01-F09: Mental disorders due to physiological conditions** | -0.10 (-0.15  - -0.03) | 0.91 (0.86  - 0.97) | 100 | -0.18 (-0.49  - 0.13) | 0.84 (0.61  - 1.14) | 0.27 |
| **ICD-10 F40-F48: Anxiety and stress-related disorders** | -0.11 (-0.16  - -0.05) | 0.90 (0.85  - 0.96) | 99.98 | 0.19 (-0.04  - 0.41) | 1.21 (0.96  - 1.51) | 0.10 |
| **ICD-10 F50-F59: Behavioral syndromes associated with physiological disturbances** | -0.09 (-0.15  - -0.04) | 0.91 (0.88  - 0.97) | 99.98 | -0.28 (-0.63  - 0.07) | 0.75 (0.53 - 1.07) | 0.11 |

**Supplementary** **Table 9.** Bootstrap estimates of predictors, indexed before conception, associated with the onset of antenatal depression in downsamples. The table compares results from the bootstrap elastic net model and the single logistic regression model. For each predictor, the bootstrap elastic net model reports median β coefficients, odds ratios (ORs), 95% confidence intervals (CIs), and variable importance in projection (VIP), whereas the single logistic regression model reports β coefficients, ORs with 95% CIs, and p-values. † For categorical predictors, repeated single logistic regression estimates indicate an overall effect of the variable as coded in the model, rather than separate level-specific estimates. NE = not estimable because of complete data separation. * = statistically significant p-value; ** = statistically significant after false discovery rate (FDR) correction.

|  | **Bootstrap performance elastic net model** | | | **Bootstrap performance single logistic regression** | | |
| --- | --- | --- | --- | --- | --- | --- |
| **Predictors** | **Median β (95% CI)** | **Odds ratio (95% CI)** | **VIP** | **β (95% CI)** | **Odds Ratio (95% CI)** | **p** |
| **White ethnicity (vs other ethnic groups)†** | -0.11 (-0.18 - -0.06) | 0.90 (0.83 - 0.94) | 99.52 | 0.26 (0.17 - 0.34) | 1.29 (1.19 - 1.41) | <0.001** |
| **Single marital status (vs cohabiting/married)†** | -0.30 (-0.34 - -0.21) | 0.74 (0.71 - 0.81) | 100 | 0.45 (0.38 - 0.52) | 1.57 (1.46 - 1.69) | <0.001** |
| **Married status (vs single/cohabiting)†** | 0.27 (0.20 - 0.34) | 1.31 (1.22 - 1.40) | 100 |  |  |  |
| **Primiparity (first pregnancy)** | 0.19 (0.09 - 0.28) | 1.21 (1.09 - 1.33) | 99.98 | 0.34 (0.21 - 0.47) | 1.40 (1.23 - 1.60) | <0.001** |
| **Use of antidepressants** | 0.30 (0.18 - 0.40) | 1.35 (1.19 - 1.49) | 100 | 1.50 (1.31 - 1.70) | 4.50 (3.70 - 5.48) | <0.001** |
| **Depressive symptom severity** | 0.45 (0.21 - 0.69) | 1.56 (1.24 - 2.00) | 99.98 | 0.29 (0.25 - 0.32) | 1.33 (1.29 - 1.38) | <0.001** |
| **ICD-10 F10-F19: Substance use disorders** | -0.17 (-0.26 - -0.07) | 0.84 (0.77 - 0.93) | 100 | -0.21 (-0.61 - 0.19) | 0.81 (0.54 - 1.21) | 0.31 |
| **ICD-10 F20-F29: Psychotic disorders** | 0.17 (0.02 - 0.34) | 1.19 (1.02 - 1.40) | 99.7 | 2.47 (1.70 - 3.25) | 11.88 (5.47 - 25.80) | <0.001** |
| **ICD-10 F40-F48: Anxiety and stress-related disorders** | -0.18 (-0.26 - -0.08) | 0.84 (0.77 - 0.92) | 99.96 | 0.28 (-0.01 - 0.57) | 1.33 (0.99 - 1.78) | 0.06 |
| **ICD-10 F50-F59: Behavioral syndromes associated with physiological disturbances** | -0.14 (-0.22 - -0.05) | 0.87 (0.80 - 0.95) | 100 | -0.15 (-0.58 - 0.29) | 0.86 (0.56 - 1.33) | 0.51 |
| **ICD-10 H60-H95: Ear and mastoid process diseases** | 0.16 (0.09 - 0.25) | 1.17 (1.09 - 1.28) | 99.96 | NE | NE | NE |
| **ICD-10 O20-O29: Other maternal disorders related to pregnancy** | 0.15 (0.04 - 0.22) | 1.16 (1.04 - 1.25) | 99.96 | 0.18 (-0.12 - 0.47) | 1.19 (0.89 - 1.60) | 0.23 |
| **ICD-10 O85-O92: Puerperium-related complications** | 0.10 (0.02 - 0.19) | 1.11 (1.02 - 1.21) | 99.8 | 0.70 (-0.38 - 1.77) | 2.01 (0.68 - 5.88) | 0.2 |
| **ICD-10 Z77-Z99: Factors influencing health status** | -0.13 (-0.20 - -0.02) | 0.88 (0.82 - 0.98) | 99.92 | -0.16 (-0.37 - 0.04) | 0.85 (0.69 - 1.04) | 0.11 |

**Supplementary Table 10.** Bootstrap estimates of predictors associated with the onset of postnatal depression in downsamples. Except for smoking during pregnancy, all other predictors were indexed before conception. The table compares results from the bootstrap elastic net model and the single logistic regression model. For each predictor, the bootstrap elastic net model reports median β coefficients, odds ratios (ORs), 95% confidence intervals (CIs), and variable importance in projection (VIP), whereas the single logistic regression model reports β coefficients, ORs with 95% CIs, and p-values. Positive coefficients indicate increased odds of postnatal depression onset, whereas negative coefficients indicate reduced odds. † For categorical predictors, repeated single logistic regression estimates indicate an overall effect of the variable as coded in the model, rather than separate level-specific estimates. * = p-value significant; ** = p-value significant after false discovery rate (FDR) correction.

|  | **Bootstrap performance elastic net model** | | | **Bootstrap performance single logistic regression** | | |
| --- | --- | --- | --- | --- | --- | --- |
| **Predictors** | **Median β (95% CI)** | **Odds ratio (95% CI)** | **VIP** | **β (95% CI)** | **Odds Ratio (95% CI)** | **p** |
| **White ethnicity (vs other ethnic groups)†** | -0.15 (-0.24 - -0.10) | 0.86 (0.78 - 0.90) | 99.98 | 0.29 (0.21 - 0.37) | 1.34 (1.23 - 1.45) | <0.001** |
| **Single marital status (vs cohabiting/married)†** | -0.35 (-0.44 - -0.29) | 0.71 (0.64 - 0.75) | 100 | 0.46 (0.39 - 0.53) | 1.58 (1.48 - 1.69) | <0.001** |
| **Married status (vs single/cohabiting)†** | 0.25 (0.14 - 0.30) | 1.28 (1.15 - 1.34) | 100 |  |  |  |
| **Index of Multiple Deprivation** | 0.08 (0.01 - 0.14) | 1.08 (1.01 - 1.15) | 99.12 | 0.01 (0.00 - 0.01) | 1.01 (1.00 - 1.01) | <0.05** |
| **Primiparity (first pregnancy)** | 0.17 (0.08 - 0.25) | 1.18 (1.08 - 1.29) | 100 | 0.35 (0.22 - 0.47) | 1.41 (1.25 - 1.60) | <0.001** |
| **Use of antidepressants** | 0.20 (0.09 - 0.29) | 1.22 (1.10 - 1.34) | 100 | 1.34 (1.16 - 1.53) | 3.83 (3.18 - 4.62) | <0.001** |
| **Depressive symptom severity** | 0.31 (0.22 - 0.55) | 1.37 (1.24 - 1.73) | 99.98 | 0.27 (0.24 - 0.30) | 1.31 (1.27 - 1.35) | <0.001** |
| **ICD-10 F10-F19: Substance use disorders** | -0.13 (-0.20 - -0.04) | 0.88 (0.82 - 0.96) | 99.98 | -0.09 (-0.47 - 0.29) | 0.91 (0.62 - 1.33) | 0.63 |
| **ICD-10 F40-F48: Anxiety and stress-related disorders** | -0.10 (-0.17 - -0.03) | 0.90 (0.84 - 0.97) | 99.72 | 0.29 (0.02 - 0.56) | 1.34 (1.02 - 1.76) | <0.05** |
| **ICD-10 F50-F59: Behavioral syndromes associated with physiological disturbances** | -0.10 (-0.16 - -0.02) | 0.91 (0.85 - 0.98) | 99.76 | -0.21 (-0.64 - 0.22) | 0.81 (0.53 - 1.24) | 0.33 |
| **ICD-10 Z69-Z76: Other circumstances related to health service contact** | -0.08 (-0.16 - 0.00) | 0.93 (0.85 - 1.00) | 98.58 | -0.45 (-0.85 - -0.05) | 0.64 (0.43 - 0.95) | <0.05* |
| **Smoking during pregnancy** | 0.45 (0.36 - 0.53) | 1.57 (1.43 - 1.70) | 100 | 1.04 (0.81 - 1.28) | 2.84 (2.25 - 3.60) | <0.001** |

**Supplementary Table 11.** Bootstrap estimates of predictors associated with admission to acute psychiatric care or home treatment during the perinatal period in the downsamples. The table compares results from the bootstrap elastic net model and the single logistic regression model. For each predictor, the bootstrap elastic net model reports median β coefficients, odds ratios (ORs), 95% confidence intervals (CIs), and variable importance in projection (VIP), whereas the single logistic regression model reports β coefficients, ORs with 95% CIs, and p-values. Positive coefficients indicate increased odds of admission to acute psychiatric care or home treatment. * = p-value significant; ** = p-value significant after false discovery rate (FDR) correction.

|  | **Bootstrap performance elastic net model** | | | **Bootstrap performance single logistic regression** | | |
| --- | --- | --- | --- | --- | --- | --- |
| **Predictors** | **Median β (95% CI)** | **Odds ratio (95% CI)** | **VIP** | **β (95% CI)** | **Odds Ratio (95% CI)** | **p** |
| **Black African and other Black ethnicity (vs White/other)** | 0.09 (0.01 - 0.19) | 1.10 (1.01 - 1.21) | 97.16 | 0.14 (-0.01 - 0.29) | 1.15 (0.99 - 1.34) | 0.06 |
| **Use of antipsychotics** | 0.28 (0.20 - 0.39) | 1.32 (1.22 - 1.48) | 100 | 1.73 (1.40 - 2.06) | 5.64 (4.07 - 7.81) | <0.001** |
| **Use of hypnotics** | 0.11 (0.03 - 0.23) | 1.12 (1.03 - 1.26) | 98.88 | 1.51 (1.17 - 1.85) | 4.51 (3.21 - 6.34) | <0.001** |

**Supplementary Table 12.** Bootstrap estimates of predictors associated with admission to acute psychiatric care or home treatment during the antenatal period in the downsamples. The table compares results from the bootstrap elastic net model and the single logistic regression model. For each predictor, the bootstrap elastic net model reports median β coefficients, odds ratios (ORs), 95% confidence intervals (CIs), and variable importance in projection (VIP), whereas the single logistic regression model reports β coefficients, ORs with 95% CIs, and p-values. Positive coefficients indicate increased odds of admission to acute psychiatric care or home treatment during pregnancy. * = p-value significant; ** = p-value significant after false discovery rate (FDR) correction.

|  | **Bootstrap performance elastic net model** | | | **Bootstrap performance single logistic regression** | | |
| --- | --- | --- | --- | --- | --- | --- |
| **Predictors** | **Median β (95% CI)** | **Odds ratio (95% CI)** | **VIP** | **β (95% CI)** | **Odds Ratio (95% CI)** | **p** |
| **Previous inpatient admission or home treatment/crisis care** | 0.19 (0.05 - 0.39) | 1.21 (1.05 - 1.47) | 98.76 | 2.04 (1.42 - 2.67) | 7.70 (4.12 - 14.41) | <0.001** |
| **Positive symptom severity** | 0.19 (0.13 - 0.37) | 1.20 (1.14 - 1.44) | 99.78 | 0.31 (0.21 - 0.40) | 1.36 (1.24 - 1.50) | <0.001** |

**Supplementary Table 13.** Bootstrap estimates of predictors associated with admission to acute psychiatric care or home treatment during the postnatal period in the downsamples. The table compares results from the bootstrap elastic net model and the single logistic regression model. For each predictor, the bootstrap elastic net model reports median β coefficients, odds ratios (ORs), 95% confidence intervals (CIs), and variable importance in projection (VIP), whereas the single logistic regression model reports β coefficients, ORs with 95% CIs, and p-values. Positive coefficients indicate increased odds of admission to acute psychiatric care or home treatment during the postnatal period. NE = not estimable because of complete data separation. * = p-value significant; ** = p-value significant after false discovery rate (FDR) correction.

|  | **Bootstrap performance elastic net model** | | | **Bootstrap performance single logistic regression** | | |
| --- | --- | --- | --- | --- | --- | --- |
| **Predictors** | **Median β (95% CI)** | **Odds ratio (95% CI)** | **VIP** | **β (95% CI)** | **Odds Ratio (95% CI)** | **p** |
| **Previous antipsychotic use (pre-conception)** | 0.45 (0.26 - 0.65) | 1.58 (1.29 - 1.91) | 100 | 1.78 (1.41 - 2.15) | 5.91 (4.09 - 8.55) | <0.001** |
| **ICD-10 O24: Diabetes mellitus in pregnancy. childbirth and the puerperium (HES)** | 0.05 (0.01 - 0.23) | 1.05 (1.01 - 1.26) | 82.74 | NE | NE | NE |

**Supplementary Figures**

**Supplementary Figure 1. Forest plots of predictors of depression onset in the antenatal period.**

Points indicate coefficient estimates in elastic net model and horizontal bars their 95% confidence intervals. Positive coefficients denote association with increased likelihood of depression onset, whereas negative coefficients denote association with decreased likelihood.

**Supplementary Figure 2. Forest plots of predictors of depression onset in the postnatal period.**

Points indicate coefficient estimates in elastic net model and horizontal bars their 95% confidence intervals. Positive coefficients denote association with increased likelihood of depression onset, whereas negative coefficients denote association with decreased likelihood.
